# Comprehensive analysis based on the ubiquitination- and deubiquitylation-related genes reveals the function of NEURL3 in esophageal squamous cell carcinoma

**DOI:** 10.3389/fimmu.2025.1632090

**Published:** 2025-08-21

**Authors:** Yi-Wei Lin, Hui-Er Li, Chao-Qun Hong, Zi-Ang Chen, Shu-Ping Liu, Yi-Wei Xu, Fang-Cai Wu, Yu-Hui Peng

**Affiliations:** ^1^ Department of Clinical Laboratory Medicine, Esophageal Cancer Prevention and Control Research Center, Chaoshan Branch of State Key Laboratory for Esophageal Cancer Prevention and Treatment, Cancer Hospital of Shantou University Medical College, Shantou, China; ^2^ Guangdong Esophageal Cancer Institute, Guangzhou, China; ^3^ Department of Oncological Laboratory Research, Cancer Hospital of Shantou University Medical College, Shantou, China; ^4^ Department of Radiation Oncology, Esophageal Cancer Prevention and Control Research Center, Cancer Hospital of Shantou University Medical College, Shantou, China

**Keywords:** esophageal squamous cell carcinoma, ubiquitination, deubiquitylation, nomogram, prognosis, immune microenvironment

## Abstract

**Background:**

As a highly invasive gastrointestinal malignancy, esophageal squamous cell carcinoma (ESCC) carries with its high morbidity and mortality. Accumulating evidence indicates that abnormal activation of ubiquitination and deubiquitylation has been implicated in pathophysiology of ESCC. However, rare prognostic models for ubiquitination-related genes (URGs) and deubiquitylation-related genes (DRGs) have been built up in ESCC.

**Methods:**

From training dataset GSE53624, the differentially expressed prognostic URGs and DRGs were identified to develop a prognostic signature, which was validated in GSE53622 and TCGA-ESCC dataset to show the robustness of the signature. To further confirm their prognosis value, the unsupervised clustering analysis was used to develop the molecular subtypes based on the prognostic URGs and DRGs. Differences in terms of biological function, immune status, and drug sensitivity were evaluated between high- and low-risk groups. The nomogram was constructed by combining the URGs and DRGs prognostic signature and clinical characteristics to improve prediction efficacy. Loss-of-function studies were conducted to explore the biological function of NEURL3 in ESCC.

**Results:**

The URGs and DRGs prognostic signature consisted of 11 genes and exhibited high accuracy in predicting prognosis of ESCC patient. Based on these 11 URGs and DRGs, two molecular subtypes of ESCC (C1 and C2) were identified, of which C2 subtype had significantly shorter overall survival time than that of C1 subtype. The function enrichment analysis showed that these genes play key roles in essential processes such as tumor metastasis and immune response. Moreover, the risk score was closely related to infiltration abundance of some types of immune cells, gene markers of immune cells and immune checkpoint-related markers. The drug sensitivity analysis showed that dacomitinib and talazoparib may serve as anti-ESCC drugs through targeting MAPK14. The nomogram was established by combining the URGs and DRGs signature with age and TNM stage, and it also showed enhanced prognostic predictive accuracy. The *in vitro* experiments showed that knockdown of NEURL3 inhibited the proliferation and motility of ESCC cells.

**Conclusions:**

Based on the URGs and DRGs prognostic signature, a novel nomogram was constructed that could serve as a potentially reliable prognostic model and provide theoretical basis for uncovering potential therapeutic target in the treatment of ESCC.

## 1 Introduction

Esophageal cancer (EC) is a kind of gastrointestinal tumor which is highly invasive and prone to metastasis and recurrence. According to the data released by Global Cancer database, the global incidence of esophageal cancer ranks 11th, and the mortality rate ranks 7th (1). In China, the main pathological type of EC is esophageal squamous cell carcinoma (ESCC), ranking 7th in incidence and 5th in mortality (2). At present, under the combined treatments involving surgery, radiotherapy and chemotherapy, the prognosis of ESCC patients is still poor with a five-year survival rate of less than 20% (3). Therefore, it is urgent to identify some pivotal genes for predicting prognosis and serving as novel therapeutic targets for ESCC.

Recently, there has been growing evidence that post-translational modification (PTM) is one of the key mechanisms for tumorigenesis, such as ubiquitination and deubiquitylation (4). Ubiquitination and deubiquitylation are important physiological processes to determine the fate of substrates, either inducing the specific degradation of multiple proteins, as in most cases, or altering their interactions, localization, or enzymatic activities (5). Most cellular biological mechanisms involve the ubiquitination proteasome pathway to regulate DNA damage repair, participate in aging cell differentiation, affect tumor malignant transformation and mediate drug resistance (6). For example, USP5 is regarded to serve as a novel deubiquitinase for the protooncogene c-Myc, providing a mechanism governing the fate of c-Myc in hepatocellular carcinoma (7). In myelodysplastic neoplasm, the differential expression of USP15-USP7 axis and UBE2T may predict pathogenesis and poor prognosis of patients (8). USP9X is involved in multiple critical cellular processes, vital molecular pathways and specific immunological regulations in tumor microenvironment (TME) (9). GPRC5A inhibited the ubiquitination-dependent degradation of LAMTOR1 to promote docetaxel-resistance and liver metastasis (10). Furthermore, a large amount of literature proves that ubiquitinase and deubiquitinase play a crucial role in the occurrence and development of ESCC, such as USP3 (11), USP13 (12), PSMD14 (13), and USP21 (14). However, most research often explored individual ubiquitinase or deubiquitinase but has not yet employed a systematic approach, which impedes the expedition of effective prognostic and therapeutic strategies in ESCC. Given the important role of ubiquitination and deubiquitylation in cancer biology, and the potential strengths of multi-omics analyses to provide a more nuanced understanding of biological systems (15, 16), the comprehensive analysis of ubiquitination-related genes (URGs) and deubiquitylation-related genes (DRGs) in ESCC should be conducted by integrating differential expression analysis, prognostic evaluation and functional enrichment across different datasets.

In this study, to explore the link between URGs and DRGs and the prognosis of ESCC patients, we used cox proportional hazards regression and least absolute shrinkage and selection operator (LASSO) regression analyses to identify and construct the URGs and DRGs prognosis signature. Based on the prognostic URGs and DRGs, two molecular subtypes of ESCC (C1 and C2) were identified, with C2 subtype having significantly shorter overall survival (OS) time than C1 subtype. The prognostic signature was analyzed for their potential biological pathways using functional enrichment analysis. Additionally, we found the different immune statuses between low-risk and high-risk groups, and the drugs that might be used to treat the ESCC patients were also identified. Moreover, we integrated clinicopathological parameters and the URGs and DRGs prognosis signature to develop a novel nomogram for improving prognostic accuracy. Finally, we explored the function of NEURL3 in ESCC by knocking down its expression level and revealed that NEURL3 may serve as a potential therapeutic target.

## 2 Materials and methods

### 2.1 Data retrieval

The TCGA database TCGA-ESCC (https://portal.gdc.cancer.gov) was used to download the gene expression data (TPM) of 95 ESCC samples with corresponding patient’s clinical information. In addition, we obtained two datasets GSE53624 and GSE53622 from the GEO database (https://www.ncbi.nlm.nih.gov/geo). Among them, the GSE53624 dataset contained gene expression profiles of 119 ESCC samples and 119 matched normal tissues with complete survival information, and it was mainly used for preliminary analysis of this study. For external validation of prognostic features, we used the TCGA-ESCC dataset and GSE53622 dataset including microarray data from 60 ESCC samples.

GeneCards (http://www.genecards.org/) (17) is a searchable, integrative database that provides comprehensive, user-friendly information on all annotated and predicted human genes. Utilizing this platform, we filtered for protein-coding URGs and DRGs with a relevance score above 7.00 using the keyword “Ubiquitination” and “Deubiquitylation”. By combining these genes, we obtained a total of 977 URGs and DRGs. The gene symbols were listed in **Supplementary Data 1**. The research flowchart was shown in **Supplementary Figure S1**.

### 2.2 Development and verification of the URGs and DRGs prognostic signature

We analyzed the GSE53624 dataset using the limma R package (https://cran.r-project.org/package=limma) to identify differentially expressed genes (DEGs) with | log2 fold change | > 0.7 and *P* < 0.05. Meanwhile, the univariate cox proportional hazards regression analysis was conducted to determine the potential genes associated with prognosis of ESCC patient (*P* < 0.05) using survival R package (https://cran.r-project.org/package=survival). The gene symbols were listed in **Supplementary Data 1**. Subsequently, a total of 13 URGs and DRGs that were both DEGs and potential prognosis genes were identified using the Venn diagram. Then, we employed the LASSO regression analyses using glmnet R package (https://cran.r-project.org/package=glmnet) to further compress the number of genes and finally determined 11 URGs and DRGs to form the prognosis signature.

The risk score of each patient based on the URGs and DRGs prognosis signature was calculated by performing the survival and nomogramFormula R packsge(https://cran.r-project.org/package=nomogramFormula), and the ggrisk R package (https://cran.r-project.org/package=ggrisk) was employed to illustrate the association between risk curves and patient survival distributions. Meanwhile, the best cutoff value of the risk score was determined by cutoff R package (https://cran.r-project.org/package=cutoff). Then, we used the best cutoff value to classify ESCC patients into two groups (low- and high-risk groups). Subsequently, the OS time of two groups was compared by the Kaplan-Meier analysis and log-rank test through the survminer R package. The URGs and DRGs prognosis signature’s discrimination ability was evaluated using the concordance index (C-index) and Receiver Operating Characteristics (ROC) analysis by the survival, survminer (https://cran.r-project.org/package=survminer) and timeROC R packages (https://cran.r-project.org/package=timeROC).

### 2.3 CDF classification of molecular subgroups

We performed unsupervised clustering analysis using the ConsensusClusterPlus R package (https://www.bioconductor.org/packages/release/bioc/html/ConsensusClusterPlus.html) to develop the molecular subtypes based on the 11 URGs and DRGs. The optimal number of clusters was determined by the area under the cumulative distribution function (CDF) curve, the descending trend of CDF delta and the consistency of sample clustering, and the optimal number of clusters selected was 2. Then, we conducted the Kaplan-Meier analysis to compare the OS time of patient in two clusters using the survminer R package. Additionally, we plotted heatmap to explain the relationship between the clusters and clinical feathers of patient using the pheatmap R package. The GSVA R package (https://www.bioconductor.org/packages/release/bioc/html/GSVA.html) was used for Gene Set Variation Analysis (GSVA). Subsequently, the limma R package was applied to assess the difference of biological function and signaling pathway between two subtypes (adjusted *P* < 0.05), and the top 30 different functions and pathways were visualized using the pheatmap R package. Meanwhile, through the ggalluvial R package, we plotted Sankey plots for the relationship between patients in the low- and high-risk groups and patients with CDF subtypes.

### 2.4 Functional analysis of the URGs and DRGs prognosis signature

We performed Gene Ontology (GO) functional enrichment analysis through the David database (https://davidbioinformatics.nih.gov/), and then revealed the potential biological process, cellular component, and molecular function of the 11 prognostic URGs and DRGs. Furthermore, the underlying pathway associated with the DEGs in the high- and low-risk groups determined by the URGs and DRGs prognosis signature was enriched through the GO, Disease Ontology (DO), and KEGG analysis using BioEnricher R package (https://github.com/Zaoqu-Liu/BioEnricher). In addition, several biochemical processes of the DEGs were inferred by calculating the Kyoto Encyclopedia of Genes and Genomes (KEGG) official website signaling pathway-related gene set score based on the single-sample gene set enrichment analysis (ssGSEA) algorithm. Additionally, we also calculated and compared the Epithelial-mesenchymal transition (EMT) score and angiogenesis score of high- and low-risk groups based on the ssGSEA algorithm.

### 2.5 TME analysis and drug sensitivity analysis of the URGs and DRGs prognosis signature

This study employed the ssGSEA algorithm to accurately calculate the infiltration abundance of 28 immune cell types in each ESCC patient. Then, the correlation between risk score and immune cell’s abundance was analyzed by spearman correlation analysis. Moreover, using spearman correlation analysis, we examined the correlation between the risk score and gene markers of immune cells (18), or immune checkpoint-related markers to determine the suitability of hub genes for predicting the efficacy of immunotherapy (19). Additional, to examine the variation in immune cell infiltration between high-risk and low-risk groups, we used the TIMER, CIBERSORT-ABS, QUANTISEQ, EPIC, MCPCOUNTER, CIBERSORT and XCELL algorithms by IOBR R package (https://github.com/IOBR/IOBR).

The oncoPredict R packages (https://cran.r-project.org/package=oncoPredict) were used to assess the differences in drug sensitivity of chemotherapeutic drugs across different risk groups based on Genomics of Drug Sensitivity in Cancer (GDSC) database which provided the expression profiles and IC50 for anti-tumor drugs in more than 1000 cancer cells (20). The correlation between drug sensitivity and the risk score, as well as the expression of the 11 prognostic URGs and DRGs were calculated by Spearman correlation analysis. Additionally, using the AMDock software, the 3D structure of dacomitinib and talazoparib from PubChem database and the MAPK14 (IDENTIFIER = 1A9U) downloaded from RCSB PDB database, we performed computer molecular docking simulations to further verify the results of our drug screening.

### 2.6 Construction and validation of a nomogram for ESCC patients

The URGs and DRGs prognosis signature and clinical parameters associated with OS were selected by univariate and multivariate cox proportional hazards regression analysis. Next, A nomogram with endpoints of 1-, 3-, and 5-year OS was constructed based on these independent prognosis factors. The C-index and ROC analysis were used to evaluate the discrimination ability of nomogram. Time-dependent C-index curves were plotted by the pec package (https://cran.r-project.org/package=pec). Calibration curve was used to evaluate the average prediction probability of the nomogram by comparing observed outcomes with predicted probabilities, and decision curve analysis (DCA) was conducted to estimate the clinical utility of the nomogram. The Kaplan-Meier method and log-rank test were applied for comparing the variations in OS time of different risk groups.

### 2.7 Cell culture and treatment

KYSE30 and KYSE150 cells were maintained in RPMI-1640 medium (Thermo Fisher Scientific) supplemented with 10% fetal bovine serum (GIBCO) and 1% penicillin/streptomycin (C100C5, NCM Biotech). Cells were routinely cultured at 37°C in a humidified atmosphere containing 5% CO_2_. The single siRNA oligonucleotides targeting human NEURL3 (GenePharma) and negative control siRNA were transfected by using Lipofectamine™ RNAiMAX (13778150, Life Technologies).

### 2.8 Western blotting

Cells were lysed in RIPA lysis buffer (R0010, Solarbio) supplemented with Protease and Phosphatase Inhibitor Cocktail (P002, NCM Biotech). The protein sample was separated by sodium dodecyl sulfate-polyacrylamide gel electrophoresis (SDS-PAGE) and transferred to polyvinylidene difluoride membranes (FFP33, Beyotime Biotechnology). After being blocked with 5% Bovine Serum Albumin V solution (A8020, Solarbio), the membranes were incubated overnight at 4°C with the following primary antibodies: anti-NEURL3 (1:1000, A20479, ABclonal) and anti-GAPDH (1:5000, HRP-60004, Proteintech). Then, the membranes were incubated with goat anti-rabbit antibody (7076, 7074, Cell Signaling Technology) at room temperature for 1 hour. Protein bands were visualized using Western blotting luminol reagent (sc-2048, Santa Cruz).

### 2.9 RT-qPCR

Total RNA was extracted from cells using the TRIzol reagent (15596018, Invitrogen). Then, the extracted RNA was reverse transcribed into cDNA with the HiScript III RT SuperMix for qPCR (+gDNA wiper) (R323, Vazyme). qPCR was performed using the ChamQ Universal SYBR qPCR Master Mix (Q711, Vazyme). The relative expression of transcripts was analyzed using the 2-ΔΔCt method. β-actin served as the internal control. All assays were performed according to the manufacturer’s instructions. The primers used in this method were displayed as following: NEURL3, F: 5’-ATGGGACCTCAGCAACAAGGCT-3’, R: 5’-AAGACCCGCCAGGCACAGTATC-3’; β-actin, F: 5’-CAACTGGGACGACATGGAGAAA-3’, R: 5’-GATAGCAACGTACATGGCTGGG-3’.

### 2.10 Cell viability assays

Twenty-four hours after transfection, the cells number was countered, and 1000 cells were seeded into 96-well plates. At specific time points, 10μl of Cell Counting Kit (CCK-8; C0039, Beyotime Biotechnology) was added to each well, and the absorbance at 450 nm was measured 2 hours later using a spectrophotometer.

### 2.11 Wound healing assay

ESCC cells were seeded into 6-well plates and then were incubated in serum-free medium for 12 hours. The sterile 200μl tip was used to draw a straight line across the cell culture, and the medium was replaced with serum-free medium. Images were acquired at specific time points using the inverted microscope, and the wound healing area was measured using ImageJ software.

### 2.12 Transwell migration and invasion assays

Transwell chambers equipped with 8.0μm pore inserts (353097, CORNING) or with a matrigel coating (354480, CORNING) were employed. Briefly, ESCC cells were suspended in serum-free medium and seeded into the upper chamber at a density of 5×10^4^ cells (migration assay) or 1×10^5^ cells (invasion assay) per well. The lower chamber was filled with medium supplemented with 10% FBS. After 48 or 72 hours of incubation, cells were fixed with 4% paraformaldehyde, stained with 0.5% crystal violet, and photographed under an inverted microscope.

### 2.13 Statistical analyses

All statistical analyses were performed using the following softwires: IBM SPSS Statistics 24, Microsoft Excel, X-tile version 3.6.1 and R version 4.2.2. The independent t-test was used for normally distributed continuous variables, while the Mann-Whitney test was employed to contrast non-normally distributed variables. Categorical variables were compared with the chi-square test or the Fisher exact test. *P* < 0.05 was considered statistically significant.

## 3 Results

### 3.1 Identification of 11 prognostic DE-URGs and DRGs

**Figure 1:**
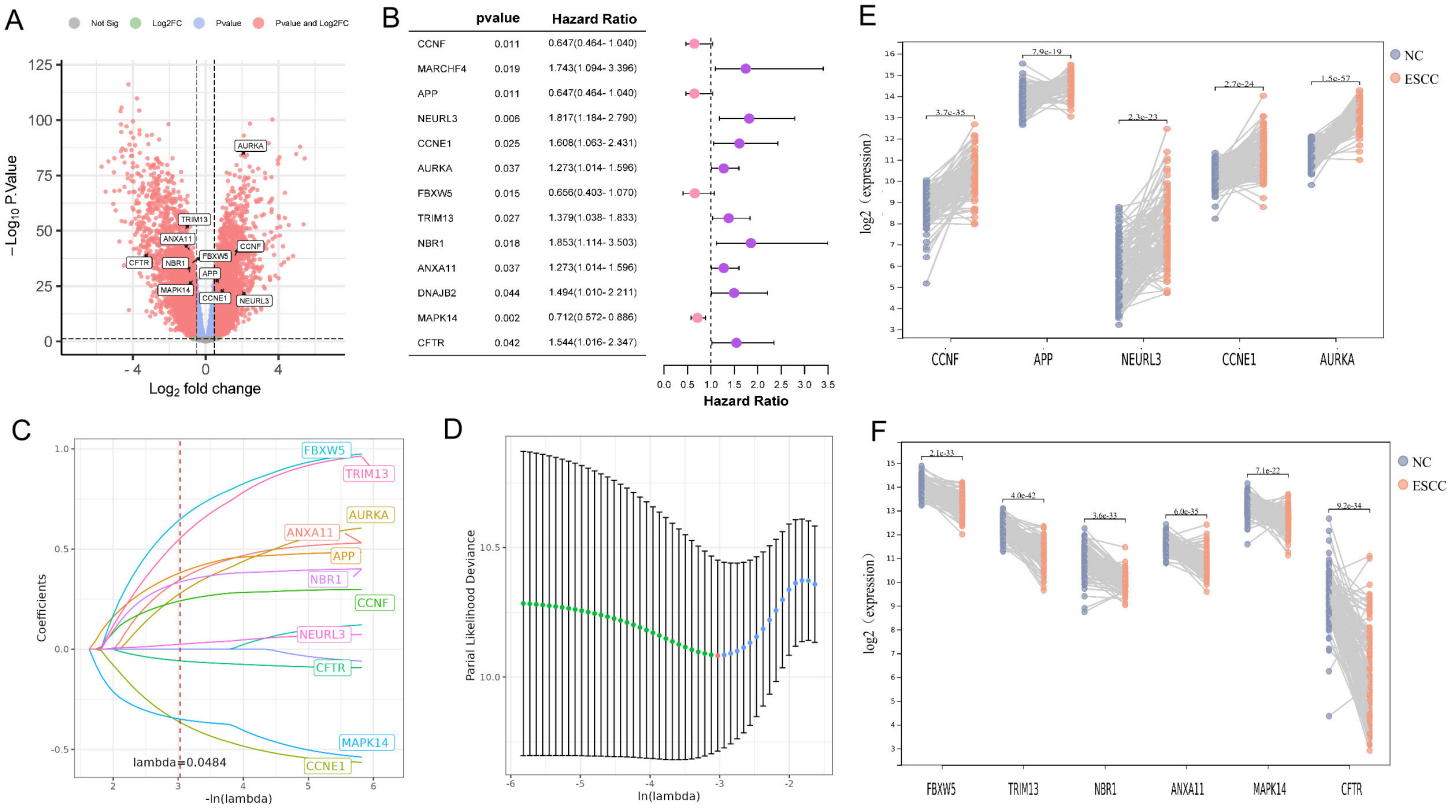
Identification of 11 prognostic DE-URGs and DRGs. **(A)** Volcano plots illustrated genes with differential expression between tumor and normal samples in the GSE53624. **(B)** The HR and 95% CI of 13 prognostic DE-URGs and DRGs based on the results of univariate cox regression analyses. **(C)** LASSO coefficient profiles (y-axis) of the gene sets and the optimal penalization coefficient (l) via 10-fold cross-validation based on partial likelihood deviance. **(D)** The dotted vertical lines represented the optimal values of l and the x-axis revealed the log (λ). **(E, F)** Visualizing the 5 up-regulated genes **(E)** and 6 down-regulated genes **(F)** between tumor and normal samples in the GSE53624.

Using the limma package, 5031 DEGs were found between the ESCC and normal groups in the training dataset GSE53624 based on the criteria of |log2 fold change| > 0.7 and *P* < 0.05 (**Figure 1A**). Meanwhile, 1709 genes associated with prognosis (*P* < 0.05) in GSE53624 were identified by the univariate cox proportional hazards regression analysis. The gene symbols were listed in **Supplementary Data 1**. A total of 13 URGs and DRGs that were both DEGs and associated with prognosis were obtained using Venn diagram (**Figure 1B**, **Supplementary Figure S2A**). Next, we conducted the LASSO analysis to remove the overfitting genes, and the URGs and DRGs prognostic signature consisting of 11 genes was created (**Figures 1C, D**, **Supplementary Figure S2B**). As shown in **Figures 1E, F**, there were 5 genes (CCNF, APP, NEURL3, CCNE1, AURKA) up-regulated, and 6 genes (FBXW5, TRIM13, NBR1, ANXA11, MAPK14, and CFTR) down-regulated in the ESCC group when compared to the normal group in GSE53624. The expression distribution of these 11 genes was plotted in the ESCC group of GSE53624 (**Supplementary Figure S2C**).

### 3.2 Development and validation of the URGs and DRGs prognostic signature

**Figure 2:**
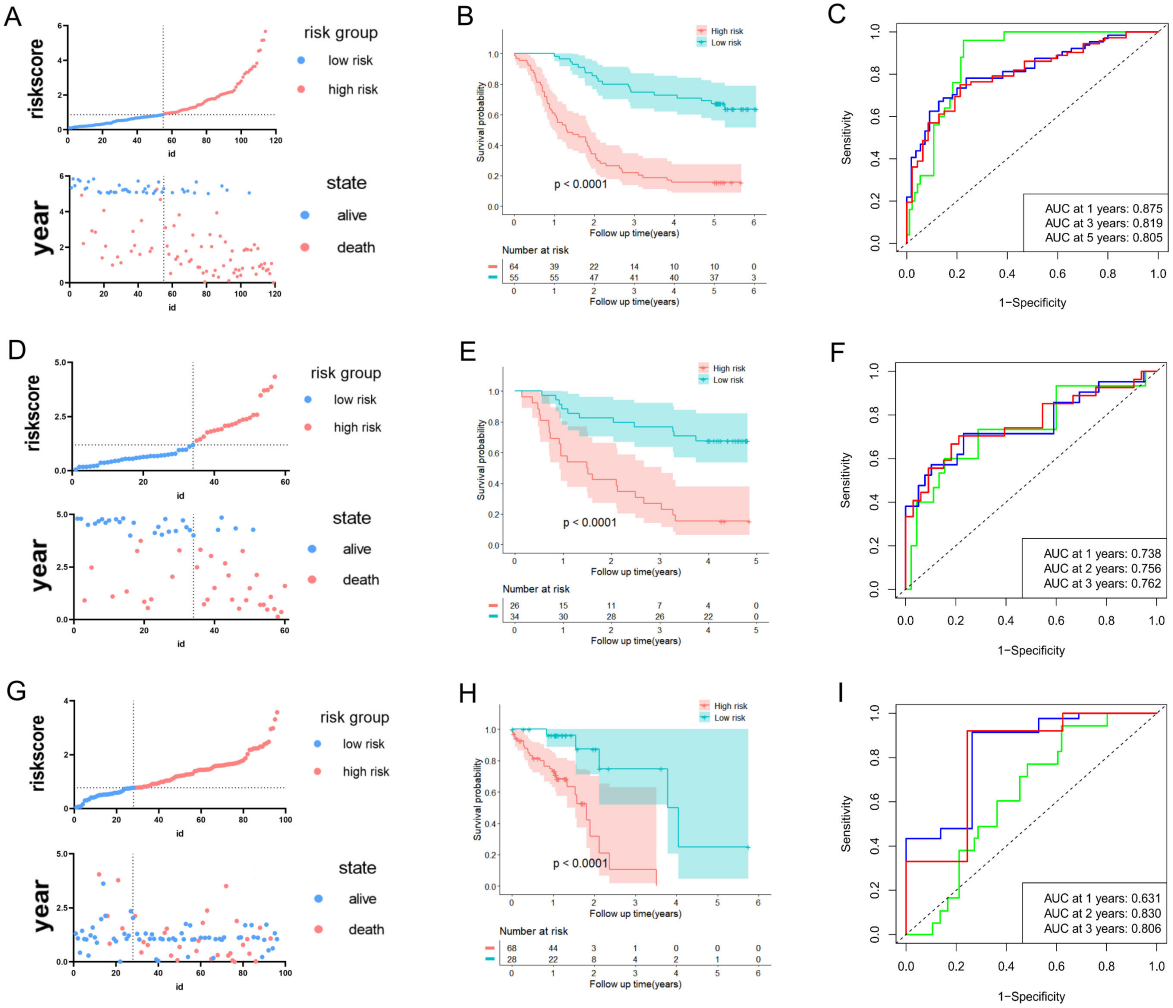
Development and validation of predictive validity of the URGs and DRGs prognostic signature. **(A)** The association between risk curves and patient survival distributions in the GSE53624. **(B)** The Kaplan-Meier curves of the high-risk and low-risk groups in the GSE53624. **(C)** The ROC curves of the URGs and DRGs prognostic signature in the GSE53624. **(D)** The association between risk curves and patient survival distributions in the GSE53622. **(E)** The Kaplan-Meier curves of the high-risk and low-risk groups in the GSE53622. **(F)** The ROC curves of the URGs and DRGs prognostic signature in the GSE53622. **(G)** The association between risk curves and patient survival distributions in the TCGA-ESCC dataset. **(H)** The Kaplan-Meier curves of the high-risk and low-risk groups in the TCGA-ESCC dataset. **(I)** The ROC curves of the URGs and DRGs prognostic signature in the TCGA-ESCC dataset.

We intended to develop a new predictive prognosis signature for ESCC based on the 11 prognostic URGs and DRGs previously identified. In training dataset GSE53624, the risk score of each patient based on the URGs and DRGs prognosis signature was calculated, and patients were classified into low- or high-risk groups as per the best cutoff value of the risk score (**Figure 2A**). The alterations in the expression of 11 prognostic URGs and DRGs between the two risk groups were illustrated in (**Supplementary Figure S3A**). Compared with patients in the low-risk group, patients in the high-risk group had worse prognosis (**Figure 2B**). Moreover, the ROC analysis was performed to evaluate the URGs and DRGs prognosis signature, which demonstrated that the AUC values for 1-, 3- and 5-year were 0.875, 0.819, and 0.805, respectively (**Figure 2C**, **Supplementary Figure S3D**). In addition, the C-index of the URGs and DRGs prognosis signature in GSE53624 was 0.699 (95% CI: 0.677-0.721) (**Supplementary Figure S3E**).

Furthermore, the predictive ability of the URGs and DRGs prognosis signature in ESCC was validated in the external validation sets (GSE53622 and TCGA-ESCC dataset). This ensures the validity and general applicability of the prognostic signature. In line with aforementioned analytical approach, the patients with ESCC in the validation datasets were also classified into low- or high-risk groups (**Figures 2D, G**). The expression of 11 prognostic URGs and DRGs changed between the high and low-risk groups in the validation datasets, as depicted in (**Supplementary Figures S3B, C**). The Kaplan-Meier analysis revealed that the low-risk individuals were consistently associated with more favorable prognosis in contrast to those at high-risk group (**Figures 2E, H**). Moreover, the URGs and DRGs prognosis signature’s AUCs of 1-, 2- and 3-year periods were 0.738, 0.756, and 0.762 in GSE53622, correspondingly, 0.631, 0.830, and 0.806 in TCGA-ESCC dataset (**Figures 2F, I**, **Supplementary Figure S3D**). The C-index of the URGs and DRGs prognosis signature were 0.685 (0.647-0.723) in GSE53622 and 0.639 (0.609-0.669) in TCGA-ESCC dataset (**Supplementary Figure S3E**). In summary, the above results indicated that the prognostic signature based on the 11 URGs and DRGs possessed remarkable discriminative ability and predictive accuracy for prognosis prediction for ESCC patient.

### 3.3 Clustering of molecular subgroup based on the 11 prognostic URGs and DRGs

**Figure 3:**
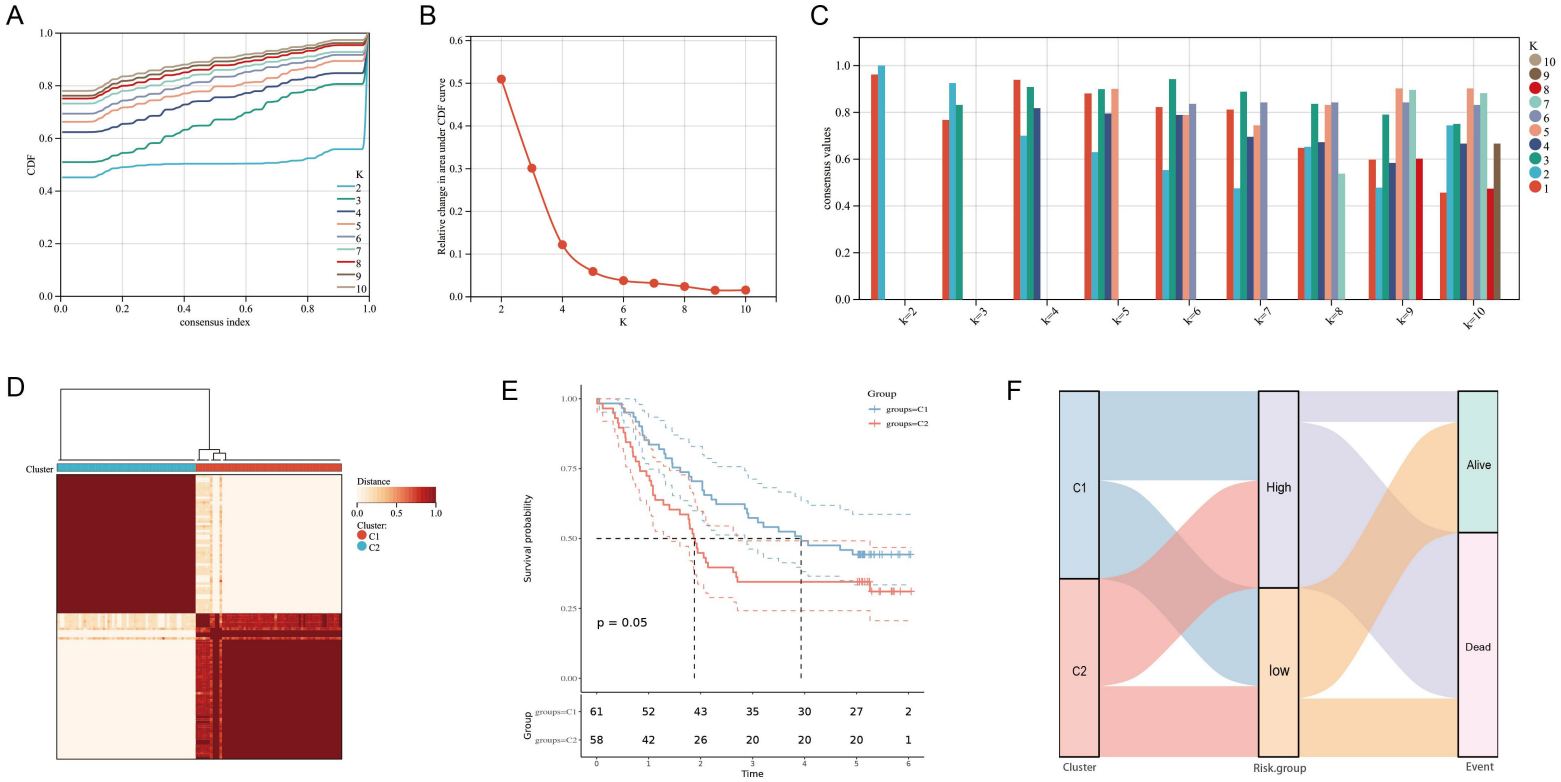
Clustering of molecular subgroup based on the 11 prognostic URGs and DRGs. **(A-C)** Cumulative distribution function curve illustrated the most effective way to cluster of these 11 genes. **(D)** The consensus score matrix of all samples when k = 2. **(E)** Kaplan-Meier curves for the overall survival time of ESCC patients among different subgroups. **(F)**Sankey diagram for two molecular subtypes and high- and low-risk groups.

Combined with the area under the CDF curve, the descending trend of CDF delta and the consistency of sample clustering, we successfully identified two subtypes based on the 11 URGs and DRGs (k = 2, C1 = 61, C2 = 58) (**Figures 3A–D**). Notably, through comparison of survival time of patient in two consensus subtypes, we observed that patients in C1 exhibited significantly better OS compared to those in C2 (*P* = 0.05) (**Figure 3E**), further confirming the prognosis value of these 11 URGs and DRGs. Additionally, we also visualized the distribution of the two subtypes of samples on clinical data in a heatmap (**Supplementary Figure S4A**). Furthermore, we utilized GSVA to calculate the functional states and signaling pathway scores of the two subtypes in GO and KEGG catalogues. Interestingly, we found that, besides the pathways related to ubiquitination, DNA replication, damage and repair pathways and immune signaling pathways were also enriched in C2 (**Supplementary Figures S4B, C**). This finding implied the reason for poor prognosis of patient in C2. Finally, we revealed the patients had a worse prognosis in both the C2 subtype and high-risk group from the result of the Sankey analysis (**Figure 3F**).

### 3.4 Functional evaluation of the URGs and DRGs prognosis signature

The GO functional enrichment analysis was conducted to investigate the biological process, cellular component, and molecular function of the 11 prognostic URGs and DRGs. We noticed that these 11 genes were involved in the cell cycle, immune response, and apoptotic process in the biological process category; cytosol, cytoplasm, and early endosome in the cellular component category; enzyme binding, cyclin-dependent protein serine/threonine kinase regulator activity, and protein kinase binding in the molecular function category (**Supplementary Figures S5A, B**).

**Figure 4:**
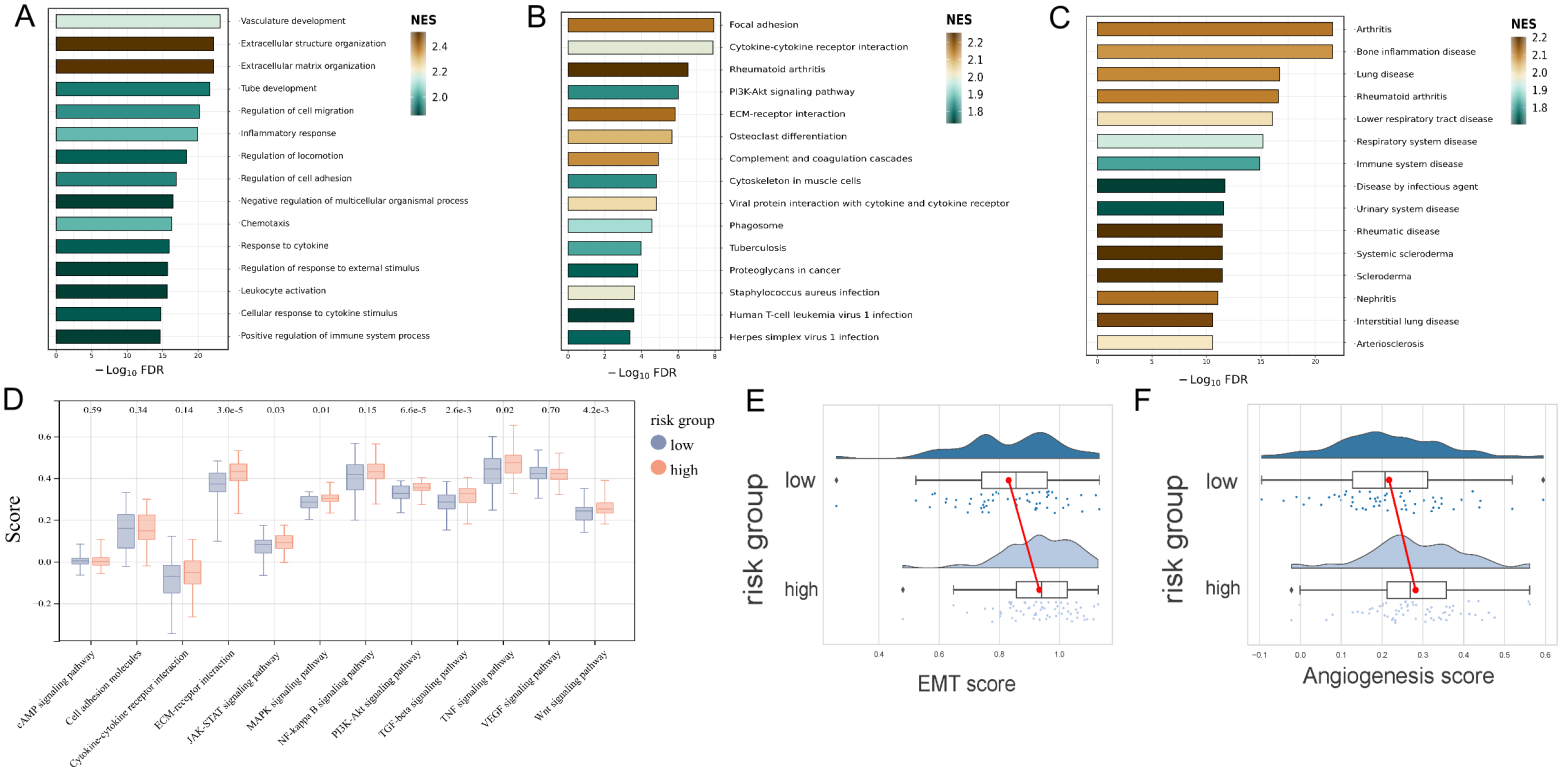
Functional evaluation of the URGs and DRGs prognosis signature. **(A)** GO analysis of the DEGs between high and low-risk groups. **(B)** KEGG analysis of the DEGs between high and low-risk groups. **(C)** DO analysis of the DEGs between high and low-risk groups. **(D)** KEGG official website signaling pathway-related gene set score analysis of the high and low-risk groups based on the ssGSEA algorithm. **(E)** EMT score analysis of the high and low-risk groups. **(F)** Angiogenesis score analysis of the high and low-risk groups.

The GO, KEGG and DO enrichment analyses were further conducted to investigate the latent biological roles of the DEGs between the high- and low-risk groups determined by the URGs and DRGs prognosis signature. Accordingly, the GO result revealed that the DEGs were prominently enriched in vasculature development, extracellular structure organization, and extracellular matrix organization (**Figure 4A**). The KEGG result suggested that the DEGs were primarily enriched in focal adhesion, cytokine-cytokine receptor interaction, and rheumatoid arthritis (**Figure 4B**). The DO result showed that the DEGs were connected with arthritis, bone inflammation disease, and lung disease (**Figure 4C**). Additionally, we calculated the KEGG official website signaling pathway-related gene set score based on the ssGSEA algorithm. The result revealed that the DEGs are significantly enriched in various signaling pathways including ECM-receptor interaction, MAPK signaling pathway, PI3K-Akt signaling pathway, TGF-beta signaling pathway, TNF signaling pathway and Wnt signaling pathway (**Figure 4D**). Furthermore, we also calculated the EMT score and angiogenesis score of two risk groups, and both of scores were all higher in high-risk group in contrast with that of the low-risk group (**Figures 4E, F**).

### 3.5 The tumor microenvironment analysis in high- and low-risk groups

**Figure 5:**
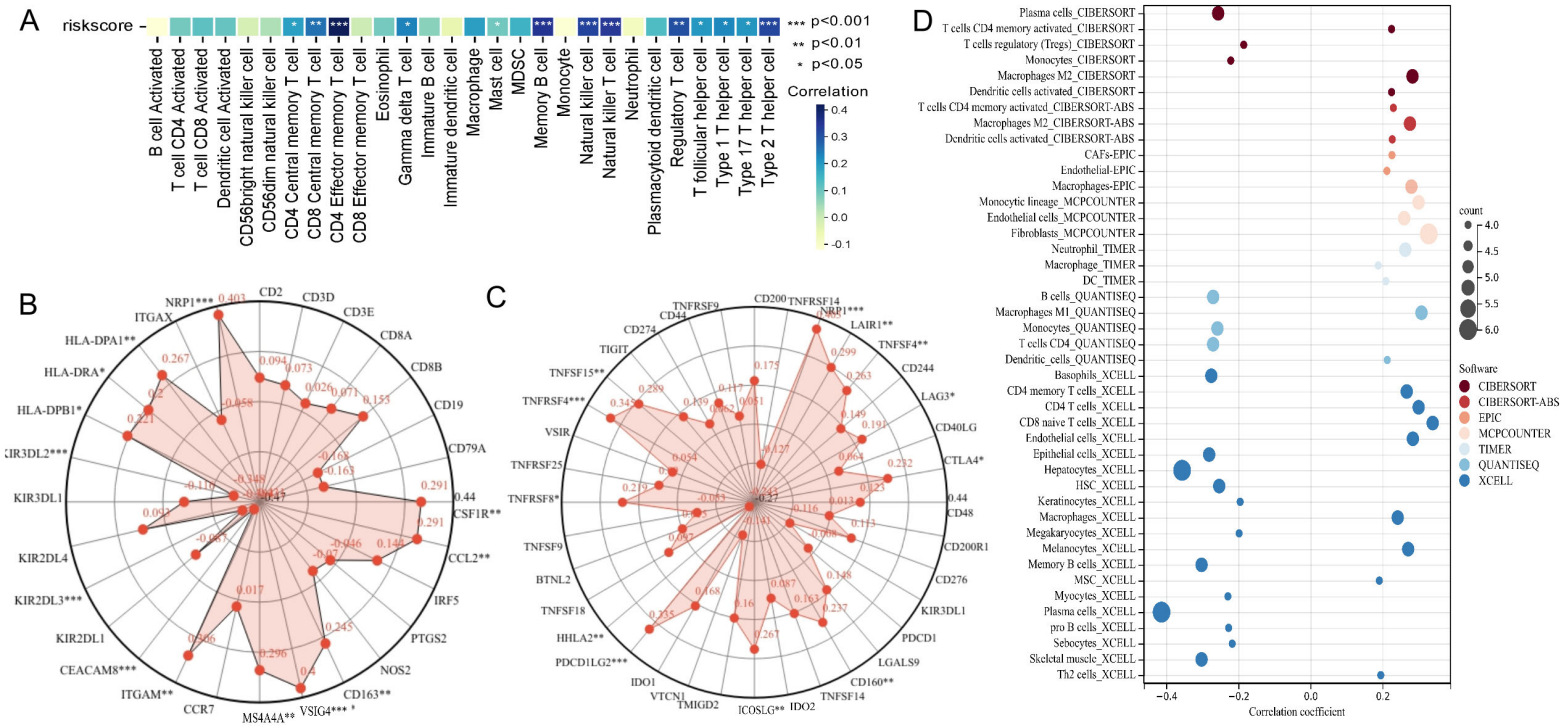
Analysis of the association between risk score of patient and immune landscape. **(A)** The correlation of risk score and 28 immune cells was calculated using the ssGSEA algorithm. **(B)** Radar plots showed the correlation of risk score and gene markers of immune cells. **(C)** Radar plots showed the correlation of risk score and immune checkpoint-related markers. **(D)** The analysis of differences of immune cell infiltration abundance between high and low-risk groups with multiple algorithms.

Given the significant impact of tumor immune microenvironment on therapeutic outcomes and patient prognosis in malignant tumors, we further investigated the potential association between risk score of patient and immune cell’s infiltration abundance. We conducted comprehensive analysis of 28 immune cell types using the ssGSEA algorithm, and the results revealed significant positive correlations between risk score and Effector memory CD4^+^ T cell, Memory B cell, Natural killer cell, Natural killer T cell, and Type 2 T helper cell (*P* < 0.001, R > 0.3) (**Figure 5A**, **Supplementary Figures S6A–E**). We further analyzed the link between risk score and the gene markers of immune cells or the immune checkpoint-related markers. The results showed a close association between risk score and CSF1R of monocytes, CCL2 of tumor-associated macrophages, CD163, VSIG4, and MS4A4A of M2 macrophages, ITGAM and CEACAM8 of neutrophils, KIR2DL3 and KIR3DL2 of natural killer cells, and HLA-DPB1, HLA-DRA, HLA-DPA1, and NRP1 of dendritic cells (**Figure 5B**), as well as closed associated with immune checkpoint markers such as NRP1, LAIR1, and TNFSF4 (**Figure 5C**). Additionally, the distinctions of immune cell levels between high- and low-risk groups were also explored through CIBERSORT, MCPCOUNTER, QUANTISEQ, EPIC, TIMER, CIBERSORT-ABS, and XCELL algorithms. As per the findings, the low-risk group had remarkably higher levels in most immune cells, including Plasma cells, regulatory T cells, Monocytes, Basophils, Memory B cells, Myocytes, pro B cells, B cells, and CD4^+^ T cells (**Figure 5D**). This may be the reason that the low-risk group has a superior prognosis.

### 3.6 Correlation analysis between drug sensitivity and URGs and DRGs prognosis signature

**Figure 6:**
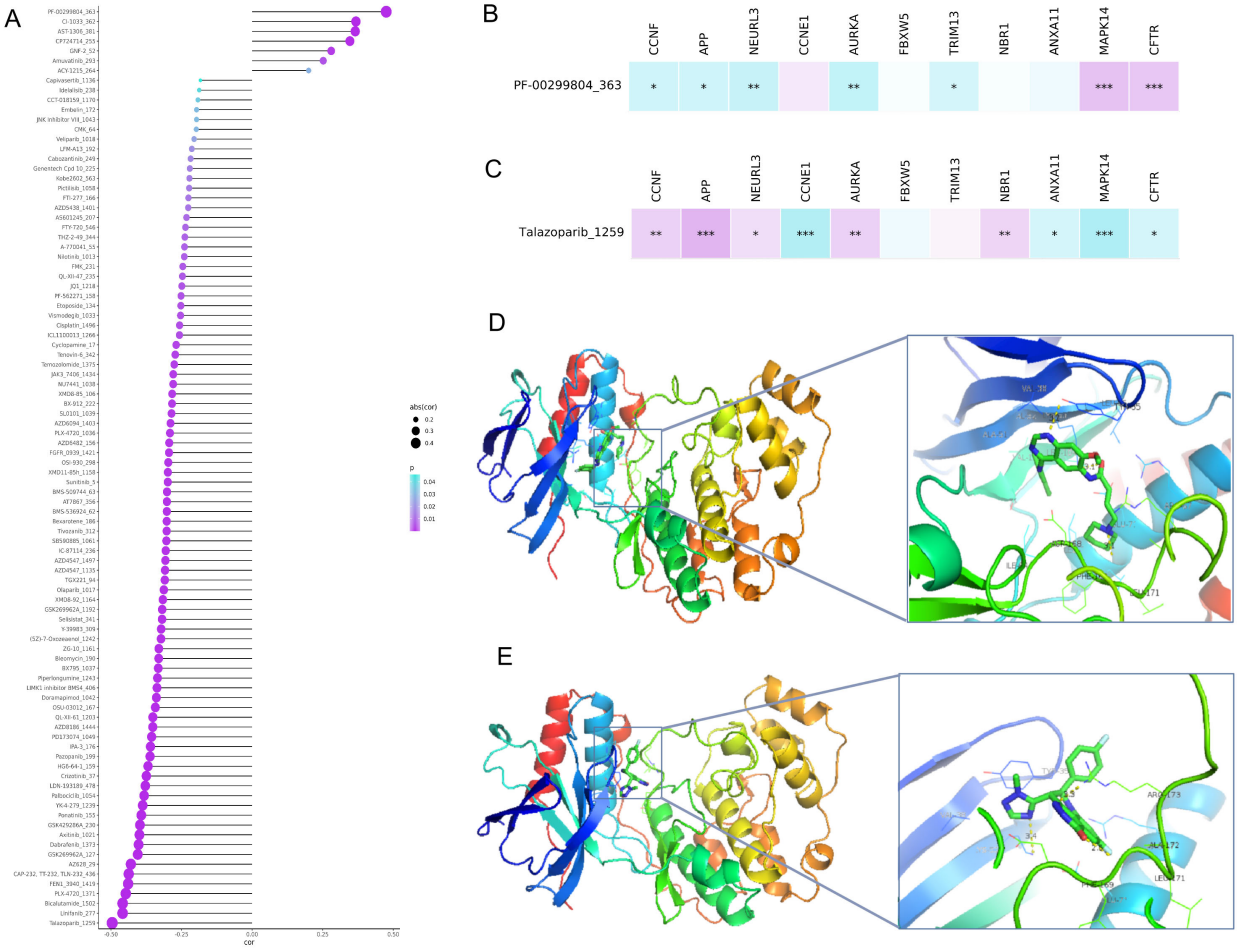
Association analysis between the URGs and DRGs prognosis signature and drug sensitivity and molecular docking between sensitive drugs and MAPK14. **(A)** Plot of correlation analysis between the risk score of ESCC patients and several drug sensitivities (IC50). **(B)** Plot of correlation analysis between the sensitivity of dacomitinib and 11 prognostic URGs and DRGs. **(C)** Plot of correlation analysis between the sensitivity of talazoparib and 11 prognostic URGs and DRGs. **(D)** Molecular docking structure diagram of MAPK14 and dacomitinib with the highest binding affinity. **(E)** Molecular docking structure diagram of MAPK14 and talazoparib with the highest binding affinity. *P < 0.05; **P < 0.01; ***P < 0.001.

To further understand the clinical application of the URGs and DRGs prognosis signature, we evaluated the association between drug sensitivity and the risk score of ESCC patients. A total of 87 drugs were negatively correlated with the risk score and were regarded as sensitive drugs for high-risk groups, while only 7 drugs showed significantly positive correlation that considered to be drug resistance to high-risk patient but might be appropriate treatment for low-risk patients (**Figure 6A**). Then, we further assessed the correlation of talazoparib and dacomitinib, of which showed the strongest negative correlation or the most prominent positive correlation, with the 11 prognostic URGs and DRGs, respectively. The results showed that the expression level of most URGs and DRGs were significantly correlated with the sensitivity to talazoparib and dacomitinib (**Figures 6B, C**, **Supplementary Figures S7A, B**). Additionally, the difference of estimated IC50 in these two drugs across different risk groups corroborated the findings of our correlation analysis (**Supplementary Figure S7C**). According to the results, as MAPK14 was most negatively correlated with talazoparib and positively correlated with dacomitinib, we selected it for molecular docking with dacomitinib and talazoparib, respectively. Based on the possible results analyzed by AMDock software, we found that both of these two drugs showed excellent binding ability with MAPK14 (**Figures 6D, E**), suggesting that they may serve as anti-ESCC drugs through targeting MAPK14.

### 3.7 Construction and validation of a nomogram for ESCC patients

**Figure 7:**
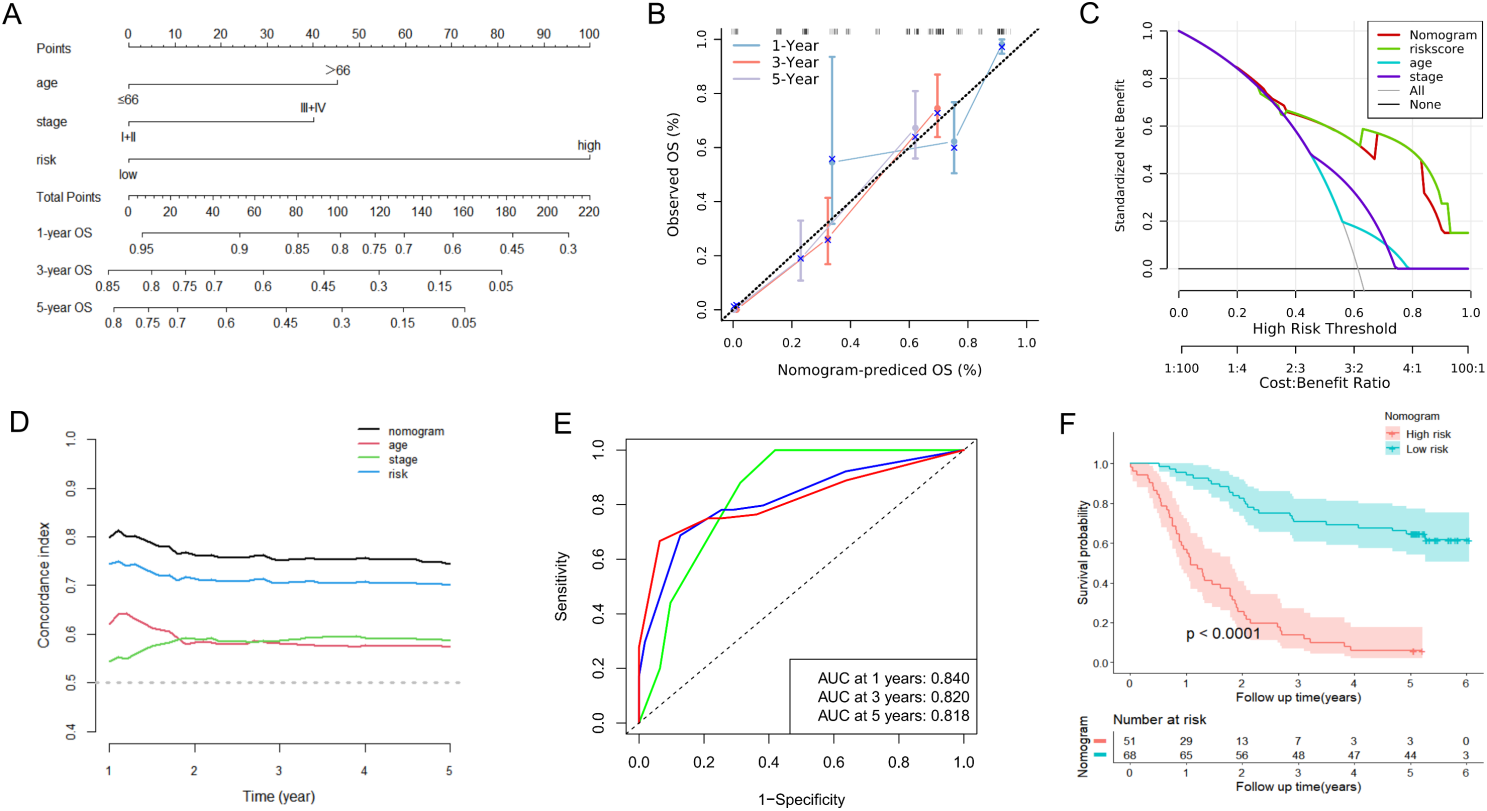
Construction and assessment of the nomogram based on the URGs and DRGs prognosis signature and clinical parameters in the GSE53624. **(A)** Nomogram integrating risk score, age and TNM stage for predicting 1-year, 3-year, and 5-year OS probabilities. **(B)** The calibration curve analysis of the nomogram for predicting 1-year, 3-year, and 5-year OS in the GSE53624. **(C)** The DCA curve analysis of the nomogram compared with risk score, age and TNM stage in the GSE53624. **(D)** The time-dependent C-index analysis of the nomogram compared with risk score, age and TNM stage in the GSE53624. **(E)** The ROC curves of the nomogram for predicting 1-year, 3-year, and 5-year OS in the GSE53624. **(F)** The Kaplan-Meier curve of the high-risk and low-risk groups determined by the nomogram in the GSE53624.

To further complement the URGs and DRGs prognosis signature’s predictive efficiency, we conducted the univariate and multivariate Cox regression analyses in training dataset GSE53624. We proved that the URGs and DRGs prognosis signature, TNM stage and age independently acted as robust prognostic markers (*P* < 0.05) (**Table 1**, **Supplementary Figures S8A, B**), which were integrated to construct for 1-, 3-, and 5-year OS nomogram (**Figure 7A**). The calibration curve analysis suggested that the predicted outcomes of the nomogram were in excellent agreement with the actual OS of ESCC patients (**Figure 7B**). The DCA curve analysis confirmed the better clinical utility of the nomogram (**Figure 7C**). The C-index of nomogram was 0.744 (95% CI: 0.721-0.767), which was higher than that of the URGs and DRGs prognosis signature (0.699, 95% CI: 0.677-0.721) (**Supplementary Figure S8H**). The time-dependent C-index analysis also showed that the nomogram had better discrimination ability in predicting survival of patients (**Figure 7D**). Moreover, the ROC curve analysis manifested that the AUC values for 1-, 3- and 5-year were 0.840, 0.820, and 0.818, respectively (**Figure 7E**). In addition, we calculated the total point of each patient and obtained the cutoff value by cutoff package. Subsequently, the ESCC patients were subdivided into low- or high-risk groups based on the cutoff value, and Kaplan-Meier analysis revealed that patients in the low-risk group exhibited a more favorable prognosis compared with high-risk group (**Figure 7F**), indicating that the nomogram can effectively determine the outlook for ESCC sufferers.

**Table 1:** Univariate and multivariate Cox regression analysis of various prognostic factors in ESCC patients.

Patient characteristics	Univariate analysis			Multivariate analysis		
	HR	95%CI	*P*	HR	95%CI	*P*
Gender (Female vs. Male)	1.210	0.685-2.138	0.511			
Age (≥66 vs. <66; y)	2.033	1.231-3.357	0.006	2.079	1.131-3.818	0.018
Drinking (Yes vs. No)	1.053	0.656-1.689	0.831			
Smoking (Yes vs. No)	0.859	0.532-1.387	0.534			
TNM stage (III-IV vs. I-II)	2.188	1.338-3.579	0.002	1.915	1.065-3.443	0.030
Tumor location (Middle vs. Lower)	0.724	0.350-1.497	0.384			
Tumor location (Upper vs. Lower)	0.643	0.329-1.255	0.196			
Tumor grade (Moderately vs. Well)	0.734	0.377-1.428	0.362			
Tumor grade (Poorly vs. Well)	0.632	0.374-1.067	0.086			
Arrhythrnia (Yes vs. No)	1.388	0.829-2.322	0.212			
Pneumonia (Yes vs. No)	1.807	0.865-3.773	0.115			
Anastomotic leak (Yes vs. No)	1.513	0.693-3.301	0.298			
Adjuvant therapy (Yes vs. No)	2.133	1.043-4.362	0.038			
Risk score (High vs. Low)	5.147	3.015-8.787	<0.001	5.563	2.871-10.780	<0.001

**Figure 8:**
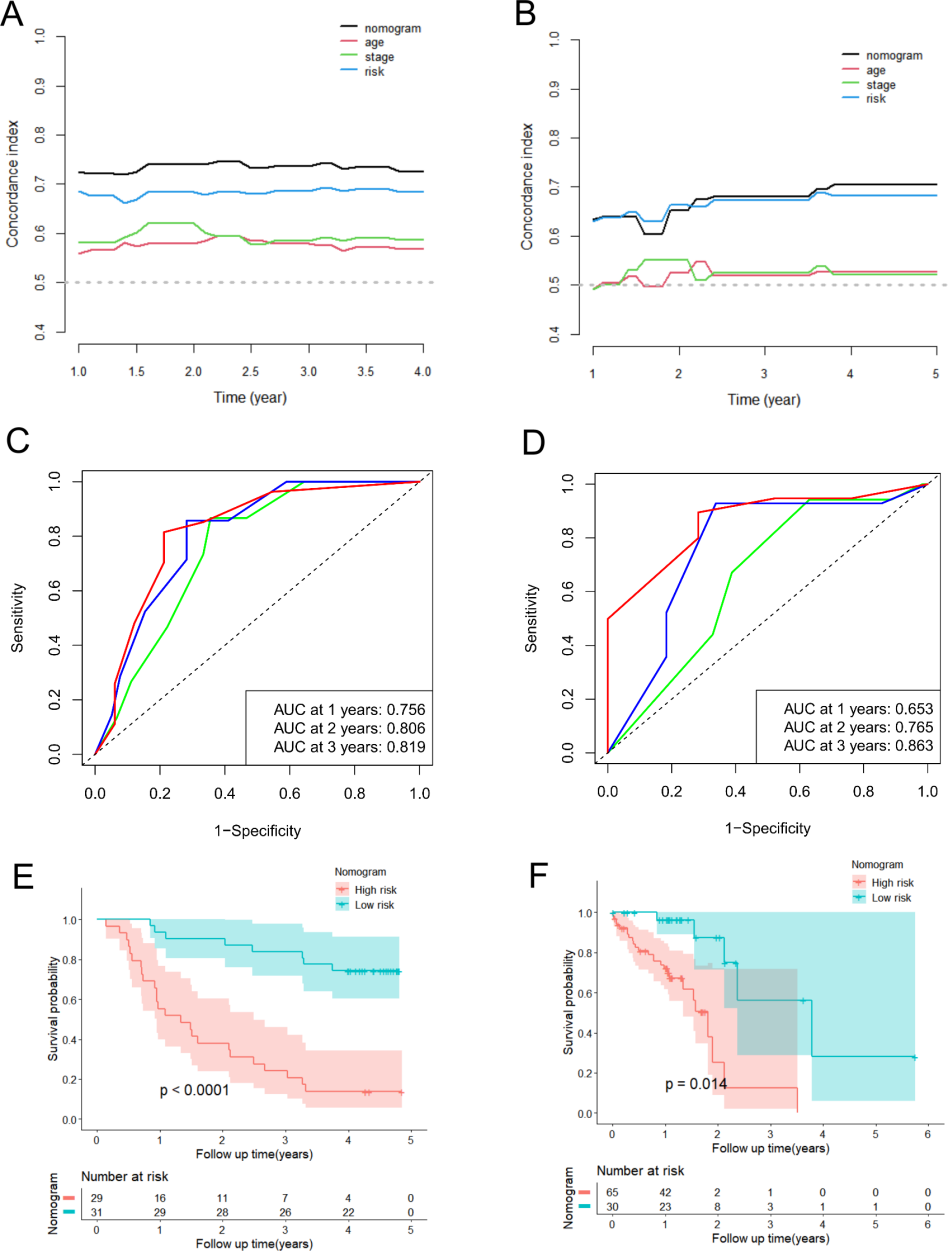
Validation of predictive efficacy of the nomogram in the validation datasets. **(A, B)** The time-dependent C-index analysis of the nomogram compared with risk score, age and TNM stage in the GSE53622 **(A)** and TCGA-ESCC **(B)** dataset. **(C, D)** The ROC curves of the nomogram for predicting 1-year, 2-year, and 3-year OS in the GSE53622 **(C)** and TCGA-ESCC **(D)** dataset. **(E, F)** The Kaplan-Meier curve of the high-risk and low-risk groups determined by the nomogram in the GSE53622 **(E)** and TCGA-ESCC **(F)** dataset.

Meanwhile, we also conducted a series of analyses mentioned above in the validation groups. As shown in **Supplementary Figures S8C, D**, the nomogram-predicted probabilities of 1-, 2-, and 3-year OS were in consistency with the actual OS, and the more outstanding clinical practical value of the nomogram were also illustrated (**Supplementary Figures S8E, F**). The C-index and time-dependent C-index analyses still demonstrated that the nomogram gave good discrimination (**Figures 8A, B**, **Supplementary Figure S8H**). The AUC values for 1-, 2- and 3-year of the nomogram were 0.756, 0.806, and 0.819, correspondingly, in GSE53622 (**Figure 8C**), and 0.653, 0.765, and 0.863 in TCGA-ESCC dataset (**Figure 8D**; **Supplementary Figure S8G**). Moreover, high-risk ESCC patients were consistently associated with poor outcomes in both validation groups (**Figures 8E, F**). These results revealed that this novel nomogram possessed tolerable accuracy and applicability for survival prediction for ESCC patients.

### 3.8 Knockdown of NEURL3 inhibited the proliferation and motility of ESCC cells

**Figure 9:**
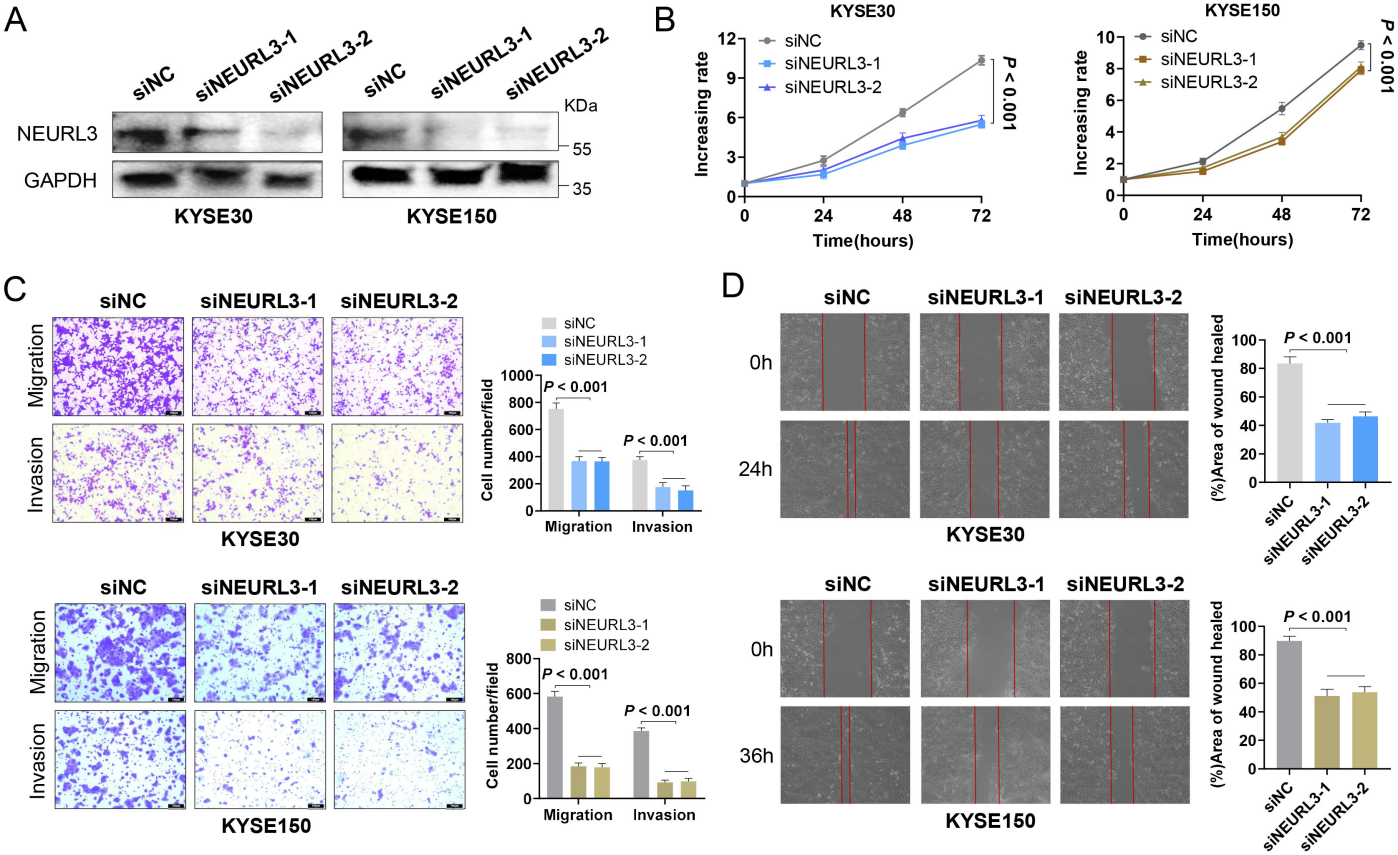
NEURL3 promotes proliferation, migration and invasion of ESCC cells. **(A)** Western blotting assay was used to show knockdown efficiency of NEURL3 in KYSE30 and KYSE150 cells by two independent siRNAs. **(B)** Proliferation ability of KYSE30 or KYSE150 cells transfected with control or siNEURL3 was measured by CCK8 assay. **(C)** Transwell assay to explore the motility of control cells compared to NEURL3 knockdown cells. **(D)** Wound healing assay to show the migration ability of control cells compared to NEURL3-depleted cells.

Among the 11 prognostic DE-URGs and DRGs, we found that NEURL3 was the upregulated expression gene with the greatest multiple of expression difference, and it was also significantly highly expressed in ESCC tissues of GSE45670, GSE53622, GSE53624 and GSE161533 (**Supplementary Figure S9A**). However, the role of NEURL3 remains unclear in ESCC. To explore the biological function of NEURL3 in ESCC, firstly, we detected the expression of NEURL3 in several ESCC cell lines including KYSE30, KYSE140, KYSE150 and KYSE510 by using RT-qPCR and Western blotting, and the results showed that NEURL3 was highly expressed in both the KYSE30 and KYSE150 cells (**Supplementary Figures S9B, C**). Then, we knocked down NEURL3 using two siRNAs in these two cell lines. **Figure 9A** and **Supplementary Figure S9D** showed that NEURL3 could be effectively silenced by two independent siRNAs. The CCK8 assay showed that NEURL3 depletion inhibited proliferation ability of KYSE30 and KYSE150 cells (**Figure 9B**). The transwell assay showed that NEURL3 knockdown reduced the migration and invasion ability of KYSE30, and the results were confirmed in KYSE150 cells (**Figure 9C**). Subsequently, in the wound-healing assay, we found that NEURL3 depletion inhibited wound closure speed in both KYSE30 and KYSE150 cells (**Figure 9D**). These results indicated that NEURL3 could promote ESCC cell proliferation and motility *in vitro*.

## 4 Discussion

Recently, growing evidence has suggested that abnormal activation of the ubiquitin-proteasome system has been implicated in the pathophysiology of various tumors. Ubiquitinase and deubiquitinase contribute to tumor progression and therapy resistance, which is regarded to be associated with the poor prognosis of ESCC (4). For example, as a deubiquitylase, USP3 stabilizes Aurora Ato to promote proliferation and metastasis of ESCC (10). RAP80 could positively regulate the stability of USP13 to promote cell proliferation of EC cells (11). PSMD14 promotes tumor metastasis through stabilizing SNAIL in ESCC (12). USP21 is regarded as mediating MOF-K257 site’s deubiquitination to promote the progression of ESCC (13). Thus, due to the important role of ubiquitination processes in ESCC biology and less understanding of ubiquitination in the ESCC prognosis, developing biomarkers from URGs and DRGs to accurately predict the prognosis of ESCC patients is highly vital and promising.

In this study, we obtained 978 URGs and DRGs from GeneCards. Then, a series of comprehensive analyses including differential analysis, univariate cox and LASSO regression analyses, were utilized to explore the prognostic value of URGs and DRGs. We identified 11 prognostic URGs and DRGs, and there were 5 genes (CCNF, APP, NEURL3, CCNE1, AURKA) up-regulated, and 6 genes (FBXW5, TRIM13, NBR1, ANXA11, MAPK14, and CFTR) down-regulated in the ESCC group. Although these genes, including CCNF, APP, CCNE1, AURKA, ANXA11, DNAJB2, MAPK14 and CFTR, do not mainly function in the ubiquitination and deubiquitylation pathway, they are all closely related with ubiquitination process and function as an important mediator in the intricate mechanisms underlying ubiquitin-proteasome system in influencing disease progression. In addition, we noted that a study by Chen et al. classified these genes as URGs and DRGs and explored their prognostic value (4), which further supports our study to identify these eight genes as URGs and DRGs. Subsequently, we constructed and evaluated the prognosis predictive efficiency of the URGs and DRGs prognostic signature for ESCC in the training and validation datasets by using the Kaplan-Meier survival curves, time-dependent ROC curves, and C-index. The results all demonstrated that the URGs and DRGs prognostic signature exhibited high accuracy in predicting patient prognosis, and that a higher risk score meant the worse prognosis for ESCC patients. In addition, by performing CDF cluster, we identified two molecular subtypes of ESCC (C1 and C2) based on the 11 prognostic URGs and DRGs, with C2 subtype having significantly shorter OS than C1 subtype, further confirming the prognosis value of these 11 URGs and DRGs.

Then, we explored the biological function and signaling pathways of the 11 prognostic URGs and DRGs to further investigate the potential molecular processes that influence ESCC patients’ survival and prognosis. It revealed that these genes were significantly enriched in various biological processes such as the cell cycle, immune response and apoptotic process, which were closely related to the occurrence and development of ESCC. Specifically, the cell cycle is a series of highly regulated steps that are orchestrated. Impaired function of the critical gatekeepers of cell cycle progression will allow unscheduled persistent cell proliferation, which is a hallmark of cancer. The dysregulation of the cell cycle has been implicated in ESCC development (21). Immune checkpoint inhibitors can improve clinical response and survival of various tumor patients through reactivating antitumor immune response, which is required for ESCC treatment (22). Evasion of apoptosis is a major contributing factor to the development of chemo- and radiotherapy resistance, resulting in the failure of ESCC treatment (23). As we can see, these above important cellular biological processes in which the 11 prognostic URGs and DRGs participate, are crucial for the progression of ESCC. Thus, these genes may be the novel therapeutic targets of ESCC, which needs further experimental evidence.

In addition, according to the results of KEGG official website signaling pathway-related gene set score analysis, the DEGs of high-risk group were significantly enriched in several signaling pathways such as TGF-beta signaling pathway, TNF signaling pathway and Wnt signaling pathway which may contribute to cancer resistance (24, 25). As we all know, tumorigenesis is a complex and dynamic process involving cell-cell and cell-extracellular matrix (ECM) interactions that allow tumor cell growth, drug resistance and metastasis (26). Especially, local invasion is a fundamental process in cancer formation, and the cell adhesion to ECM plays an important role in this process (27). In this study, based on the results of GO and KEGG analyses, we found the DEGs were enriched in the extracellular structure organization, extracellular matrix organization and focal adhesion, and they scored higher in the ECM-receptor interaction signaling pathway. Furthermore, the EMT process is a characteristic feature of most metastatic cells (28). From the result of EMT analysis, we also observed that the high-risk group had a greater EMT score. To the best of our knowledge, cancer metastasis is the major cause of cancer mortality and accounts for about 90% of cancer deaths (28). Therefore, the role of these 11 prognostic URGs and DRGs involved in tumor metastasis might be one of the reasons for the high-risk ESCC patients with poorer prognosis.

Furthermore, from the results of KEGG and DO analyses, we also noticed that the DEGs between low- and high-risk groups were associated with bone inflammation disease, cytokine-cytokine receptor interactions, MAPK and PI3K-Akt signaling pathway. Chronic inflammation is closely related to the occurrence and progression of tumor, and many studies have demonstrated that elevated levels of inflammation are associated with poor prognosis of various tumor patients (29). The cytokine and cytokine receptor interaction networks are regarded as crucial effector on inflammation as well as tumor immunology (30). The most dysregulated inflammatory pathways linked to cancer include MAPK and PI3K-Akt pathways, which lead to tumor growth, invasion, and metastasis (24). In conclusion, the abnormal activation of inflammation-related factors in the high-risk group may be another explanation for the worse prognosis outcome.

The imbalance of angiogenic regulators drives tumor angiogenesis and causes the vasculature to develop much differently in tumors than in normal tissue, which is essential for keeping the tumor alive and facilitating its growth (31). In the result of GO function analysis, the DEGs between low- and high-risk groups were primarily also enriched in vasculature development. Moreover, the high-risk group had a greater angiogenesis score than that of low-risk group. These results remind us that the poorer prognosis of the high-risk group is possibly associated with the development of tumor angiogenesis.

As a key to tumorigenesis, the TME is constituted of diverse immune cells, interstitial cells, extracellular matrix, and tumor blood vessels, which are the basis of the investigation into TME components and their potential role in either creating a favorable environment for metastatic cancer cells or transitioning into an immunosuppressive state (32). Moreover, the infiltrating immune cell levels in TME usually change with tumorigenesis and progression (26, 33). In this study, we found that ESCC patients having low risk score had remarkably higher levels in most immune cells, including Plasma cells, regulatory T cells, Monocytes, Basophils, Memory B cells, Myocytes, pro B cells, and CD4+ T cells. This may explain why the low-risk group has a superior prognosis. The results further revealed a close association between the risk score and gene markers of immune cells such as CSF1R, CCL2 and CD163, as well as immune checkpoint molecules such as NRP1, LAIR1, and TNFSF4. Therefore, the potential of the URGs and DRGs prognostic signature acting as vital biomarkers for immunotherapy response remains to be achieved.

To evaluate the potential value of the URGs and DRGs prognosis signature for guiding therapy in ESCC, we conducted drug sensitivity test and screened out 87 drugs that regarded as sensitive drugs for high-risk groups while 7 drugs that may be able to sensitize low-risk ESCC patients. Among them, talazoparib, as the poly (adenosine diphosphate-ribose) inhibitor which has shown excellent antitumor activity in patients with breast cancer (34) and small cell lung cancer (35), was negatively correlated with the risk score and might be the more valuable drug for treating high-risk ESCC patients. Additionally, Dacomitinib, a potent irreversible pan-HER inhibitor exhibiting promising efficacy in platinum-failed R/M-ESCC (36), displayed the most prominently positive correlation and might be appropriate treatment for low-risk patients. Notably, through molecular docking simulation, both two drugs showed excellent binding ability with MAPK14. It has been reported that MAPK14 served as the regulator for the expression and activity of ubiquitinase or deubiquitinase to exert its effects in the tumor’s behavior (37–39). When we applied the drugs that showed excellent binding ability with MAPK14, to inhibit the activity of MAPK14, it would also affect the ubiquitination and deubiquitylation pathway and then make the tumor cell more sensitive to the drugs, suggesting that they may serve as anti-ESCC drugs targeting MAPK14.

To better utilize the URGs and DRGs prognostic signature to guide the prognosis prediction of ESCC patients, we screened out two potential prognostic clinical characteristics (age and TNM stage) and combined with the URGs and DRGs prognostic signature to construct the nomogram for predicting 1-, 3-, and 5-year OS. According to the results of the calibration curve, Kaplan-Meier survival curve, time-dependent ROC curve and C-index analyses in the training and validation datasets, it could be found that the nomogram showed enhanced predictive accuracy and discriminative ability when compared with the URGs and DRGs prognostic signature. This indicates that the novel nomogram could act as an admirable prognosis prediction model for ESCC patients.

Finally, we selected the NEURL3, the most upregulated expressed gene among the 11 prognostic DE-URGs and DRGs, for further experimental exploration to validate the importance of these genes for ESCC progression. NEURL3 is a 262-amino-acid E3 ubiquitin ligase that contains a neuralized homology repeat domain, a RING finger structural domain, and a C-terminus (40). Recently, the ability of NEURL3 to augment host antiviral response through catalyzing K63-linked ubiquitination of interferon regulatory factor 7 has been discovered (41). Additionally, NEURL3 is shown to function as an antiviral effector against the assembly of hepatitis C virus (42). Although it has been revealed that overexpression of NEURL3 suppressed NPC cell migration, invasion, and metastasis by promoting the degradation of Vimentin through increasing its K48-linked polyubiquitination (40), the biological function of NEURL3 in the tumorigenesis and progression, especially in ESCC, has not been thoroughly investigated. In this study, we found that NEURL3 knockdown inhibited the proliferation, invasion and migration of both KYSE30 and KYSE150 cells, which seems to be different from the role of NEURL3 in NPC. It should be acknowledged that the same gene plays different role in different tumor types is crucial for understanding cellular fate decisions in cancer (43). The potential mechanisms underlying NEURL3 in promoting ESCC proliferation and motility need to be explored in the future.

Despite the promising findings our study has produced, there are several drawbacks that need to be improved. Due to the lack of data of clinical samples in real world, the vast majority of analysis results were based on the publicly available datasets, which may have a certain bias in the source of samples. Future research should include prospective validation cohorts with multicenter samples to enhance the current model’s general applicability and improve its clinical feasibility. Moreover, the biological functions and downstream mechanisms of these 11 prognostic URGs and DRGs should be further explored by various *in vivo* and *in vitro* experiments, such as CCK8 assay, transwell assay and xenograft transplantation assay. In conclusion, our study constructed a novel nomogram based on the URGs and DRGs prognostic signature that could serve as a potentially reliable prognostic model for ESCC patients, and also provides theoretical basis for uncovering potential therapeutic target in the treatment of ESCC.

## 5 Conclusions

In this study, we developed a nomogram based on the URGs and DRGs prognostic signature, which effectively predicted the prognosis of ESCC patients. The significant differences in terms of biological function, immune status, and drug sensitivity between high- and low-risk groups have been discovered by using multiple methods. We also identified that the NEURL3, an E3 ubiquitin ligase which could promote ESCC cell proliferation and motility *in vitro*, may serve as a potential therapeutic target for ESCC.

## Data Availability

The original contributions presented in the study are included in the article/**Supplementary Material**. Further inquiries can be directed to the corresponding authors.
