# Supplementary material for: Comprehensive analysis based on the ubiquitination- and deubiquitylation-related genes reveals the function of NEURL3 in esophageal squamous cell carcinoma
Source: Front Immunol. 2025 Aug 21;16:1632090. doi: 10.3389/fimmu.2025.1632090 (PMC12408286; doi:10.3389/fimmu.2025.1632090)
Supplement: Supplementary file 1 [file Table1.docx]

Supplementary Material

# Supplementary Data

**Supplementary Data 1** The description of a total of 977 protein-coding URGs and DRGs, 5031 differentially expressed genes (DEGs) with | log2 fold change | > 0.7, and 1709 potential genes associated with prognosis of ESCC patient (*P* < 0.05).

# Supplementary Figures and Tables

##
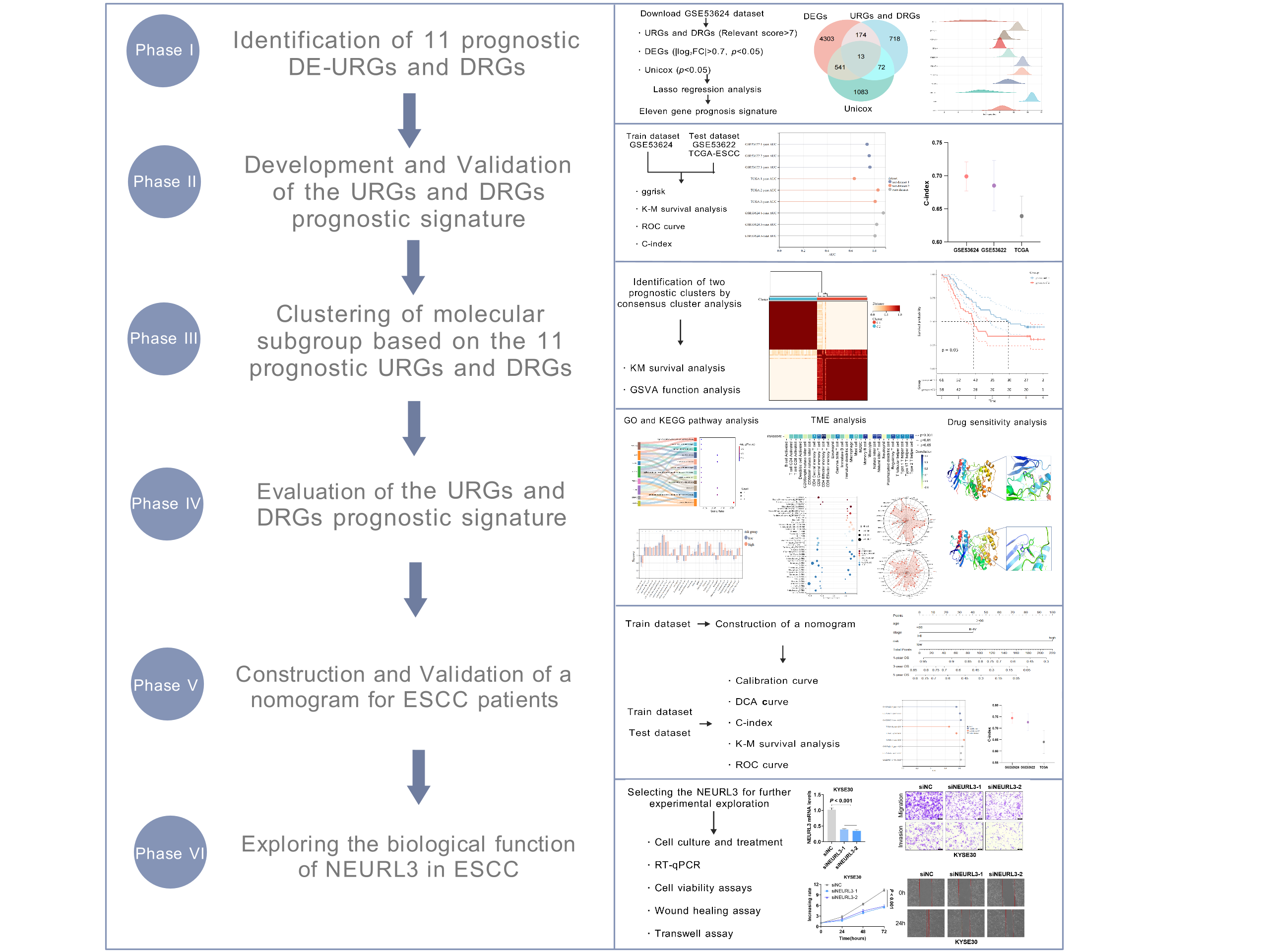
Supplementary Figures

**Fig. S1** The flowchart of the major steps in this study. C-index, concordance index; DCA, decision curve analysis; DEGs, differentially expressed genes; DRGs, deubiquitination-related genes; ESCC, esophageal squamous cell carcinoma; GO, gene ontology; KEGG, kyoto encyclopedia of genes and genomes; K-M survival analysis, Kaplan-Meier survival analysis; NEURL3, neuralized E3 ubiquitin protein ligase 3; ROC curve, receiver operating characteristic curve; TME, tumor microenvironment; URGs, ubiquitination-related genes.


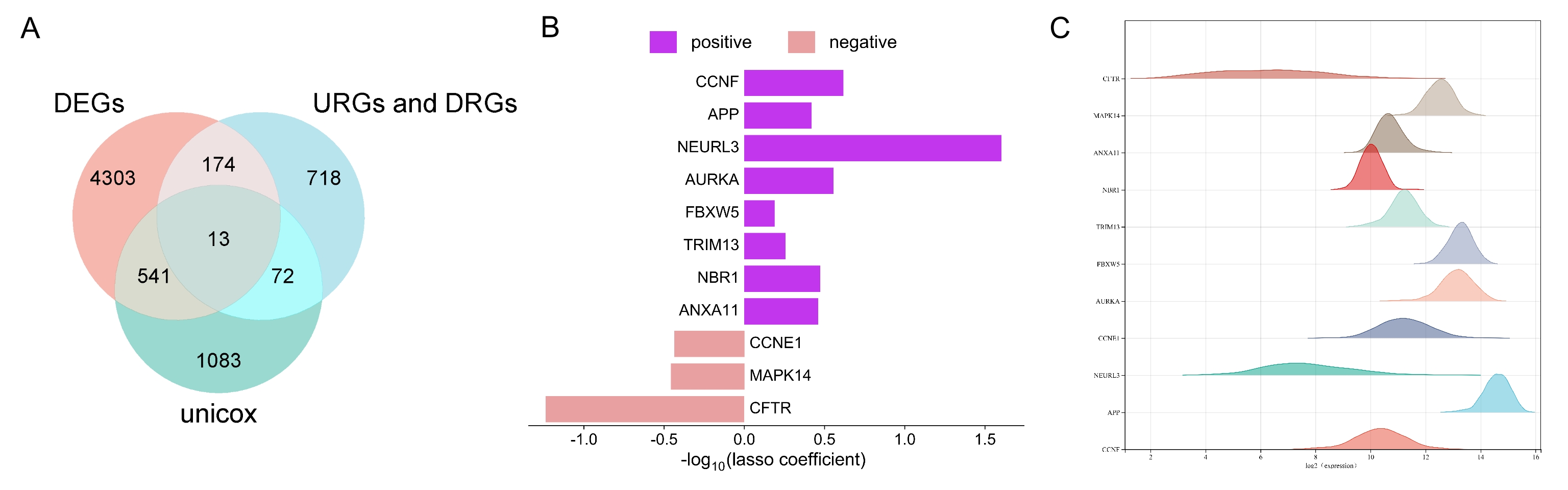


**Fig. S2** (A) Venn diagram identified common genes between DEGs, URGs and DRGs, and genes associated with prognosis. (B) Coefficients of 11 genes finally obtained from LASSO algorithm. (C) Ridgeplot plotted the expression distribution of 11 prognostic DE-URGs and DRGs in the ESCC of GSE53624.

**
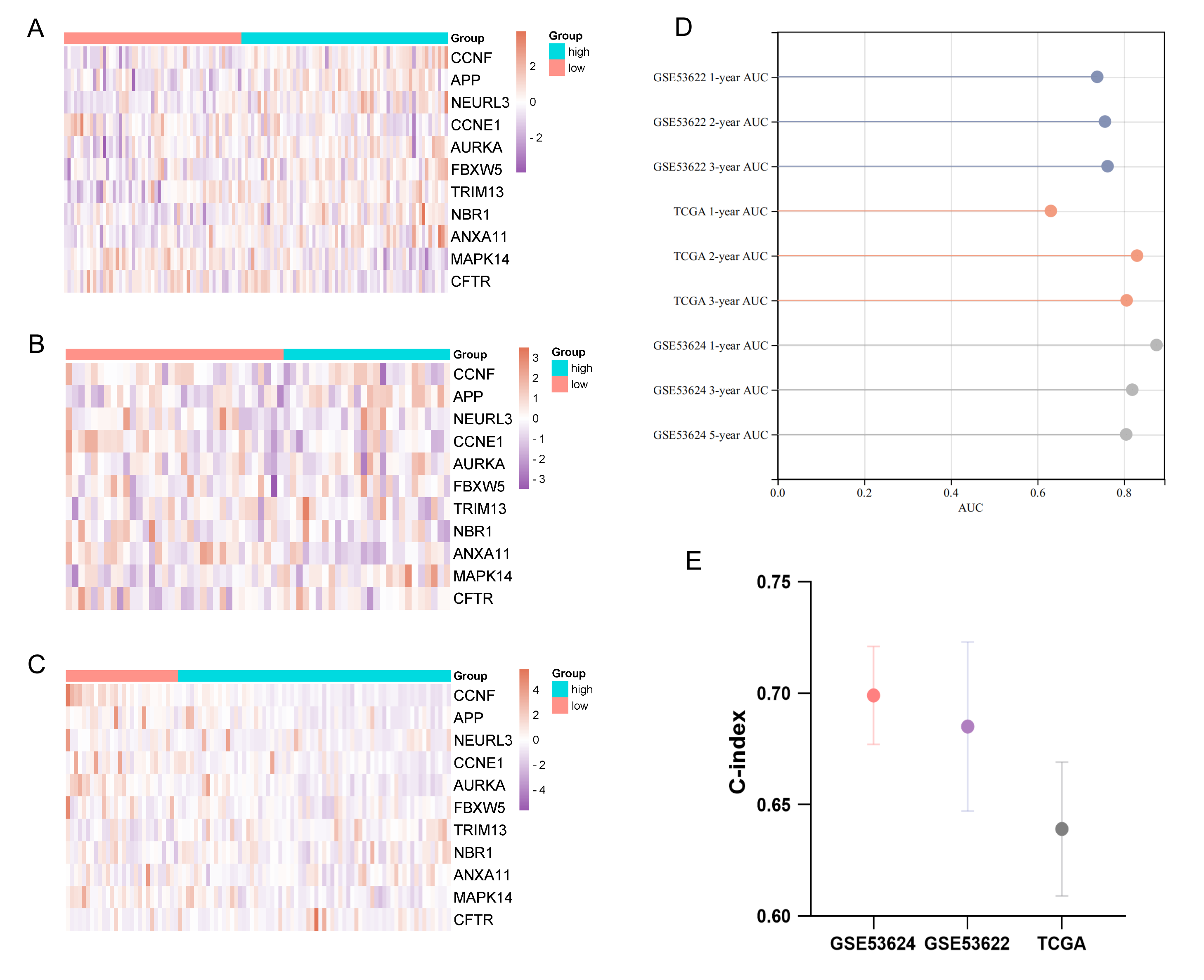
**

**Fig. S3** (A-C) A heatmap showed the distribution of 11 genes between the high-risk and low-risk groups in the GSE53624 (A), GSE53622 (B) and TCGA-ESCC (C) dataset. (D) The time-dependent AUC of the URGs and DRGs prognostic signature in the GSE53624, GSE53622 and TCGA-ESCC dataset. (E) The C-index of the URGs and DRGs prognosis signature in the GSE53624, GSE53622 and TCGA-ESCC dataset.


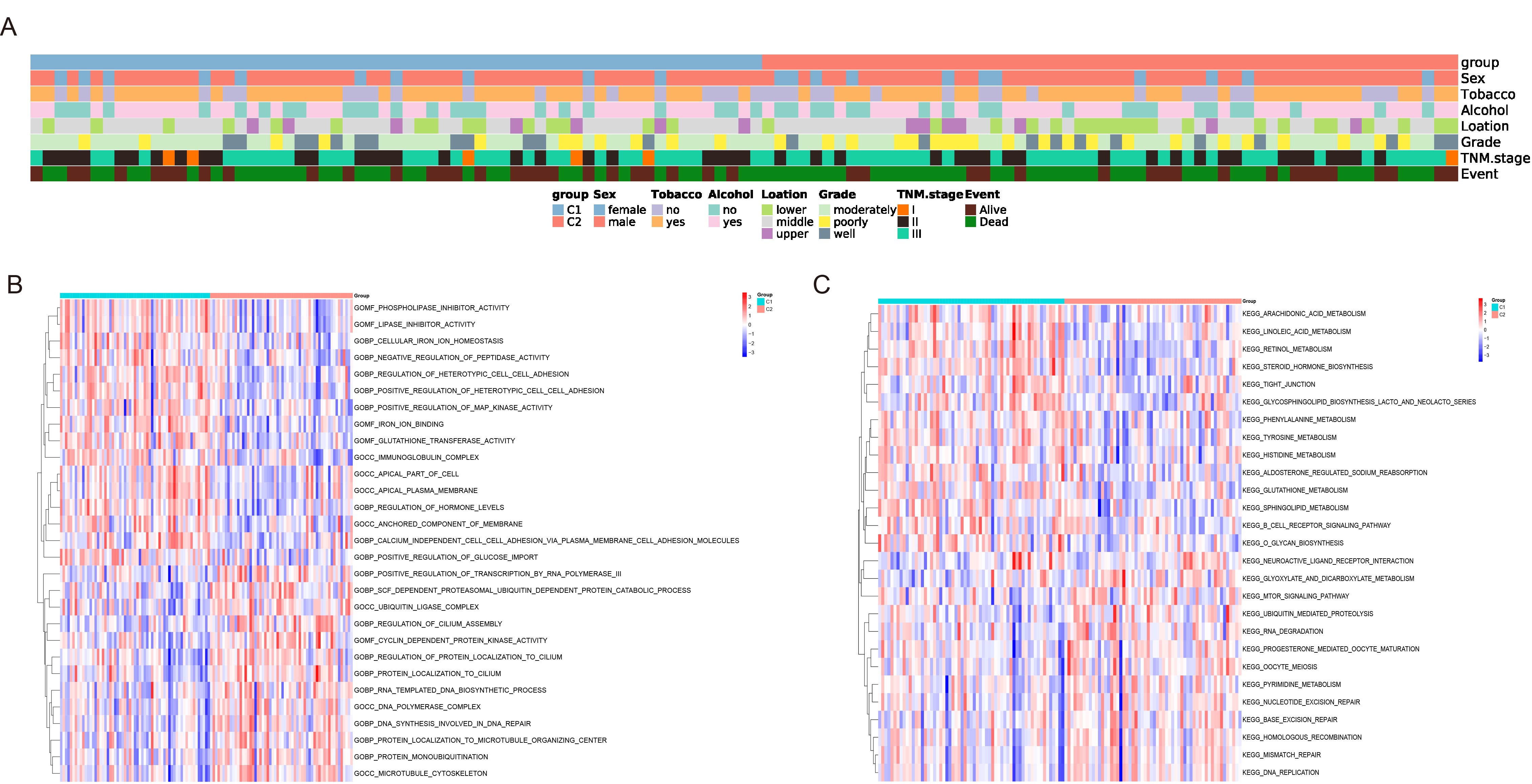


**Fig. S4** (A) A heatmap for distribution of clinical characteristics in ESCC patients with different subgroups. (B-C) A heatmap of GSVA analysis between the two subgroups in GO (B) and KEGG (C) catalogues.

**
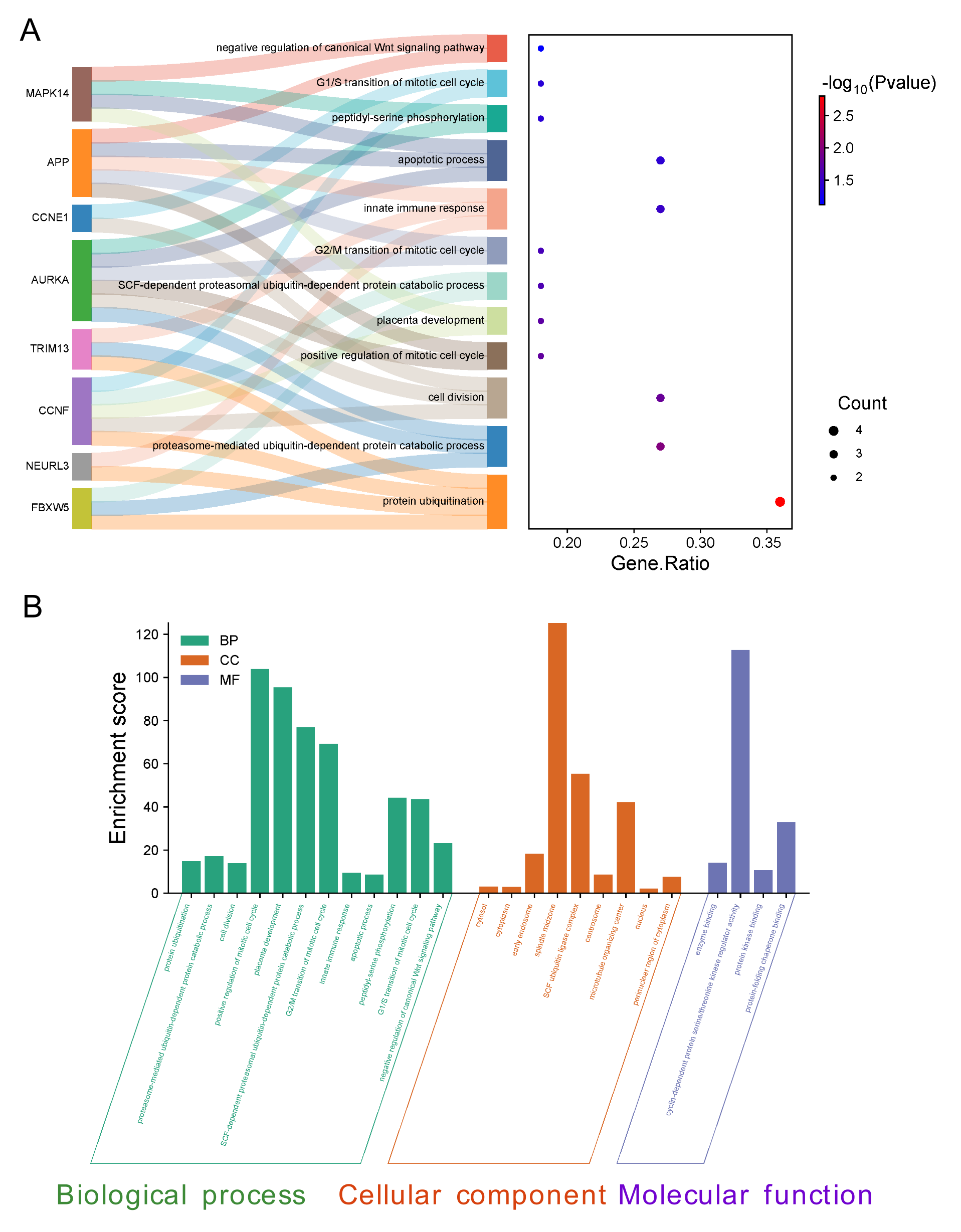
**

**Fig. S5** (A, B) GO functional enrichment analysis of the 11 prognostic URGs and DRGs.

**
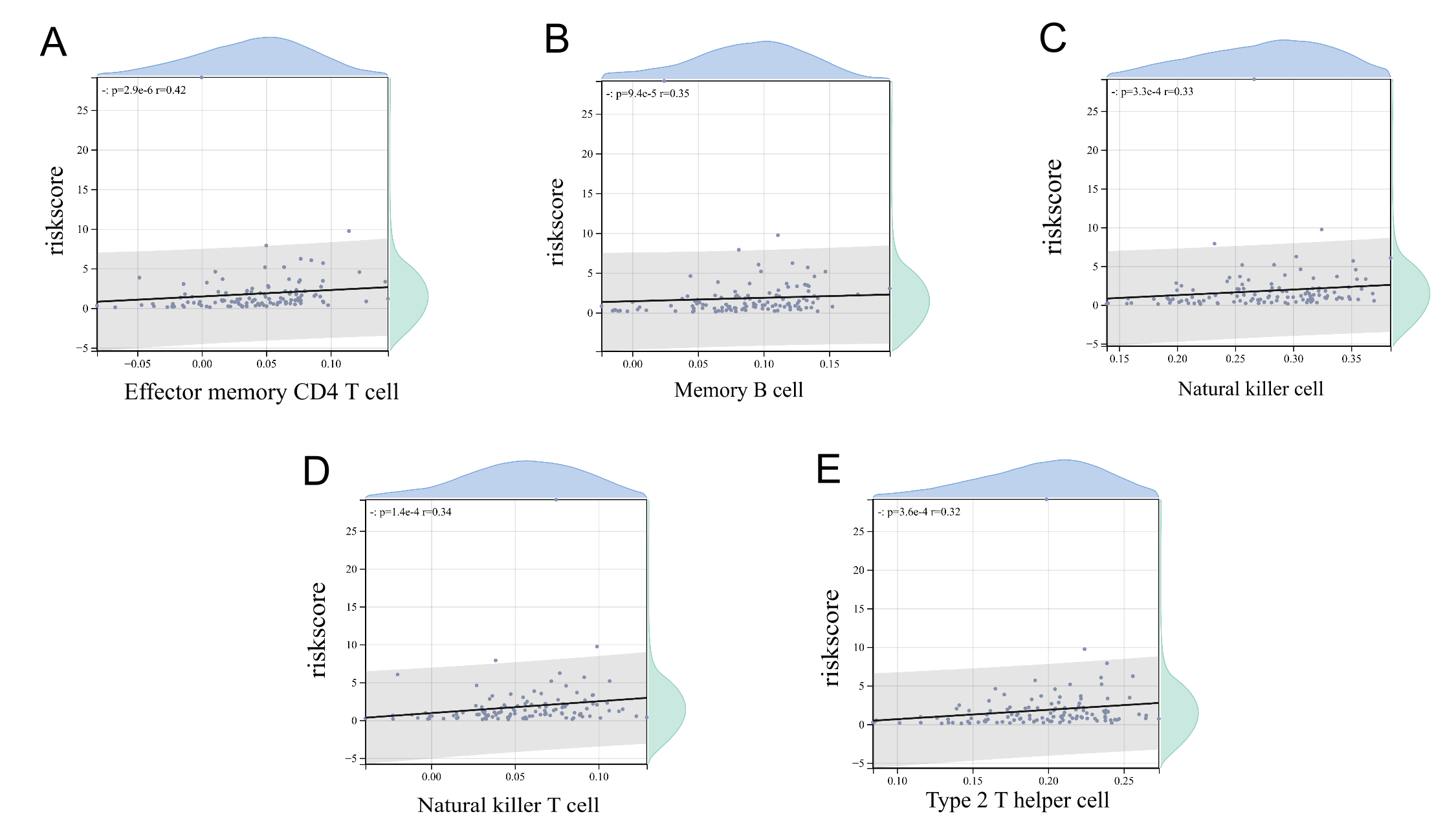
**

**Fig. S6** (A-E) Correlation analysis found that Effector memory CD4+ T cell (A), Memory B cell (B), Natural killer cell (C), Natural killer T cell (D) and Type 2 helper cell (E) had the highest correlation with risk score (P < 0.001, R > 0.3).


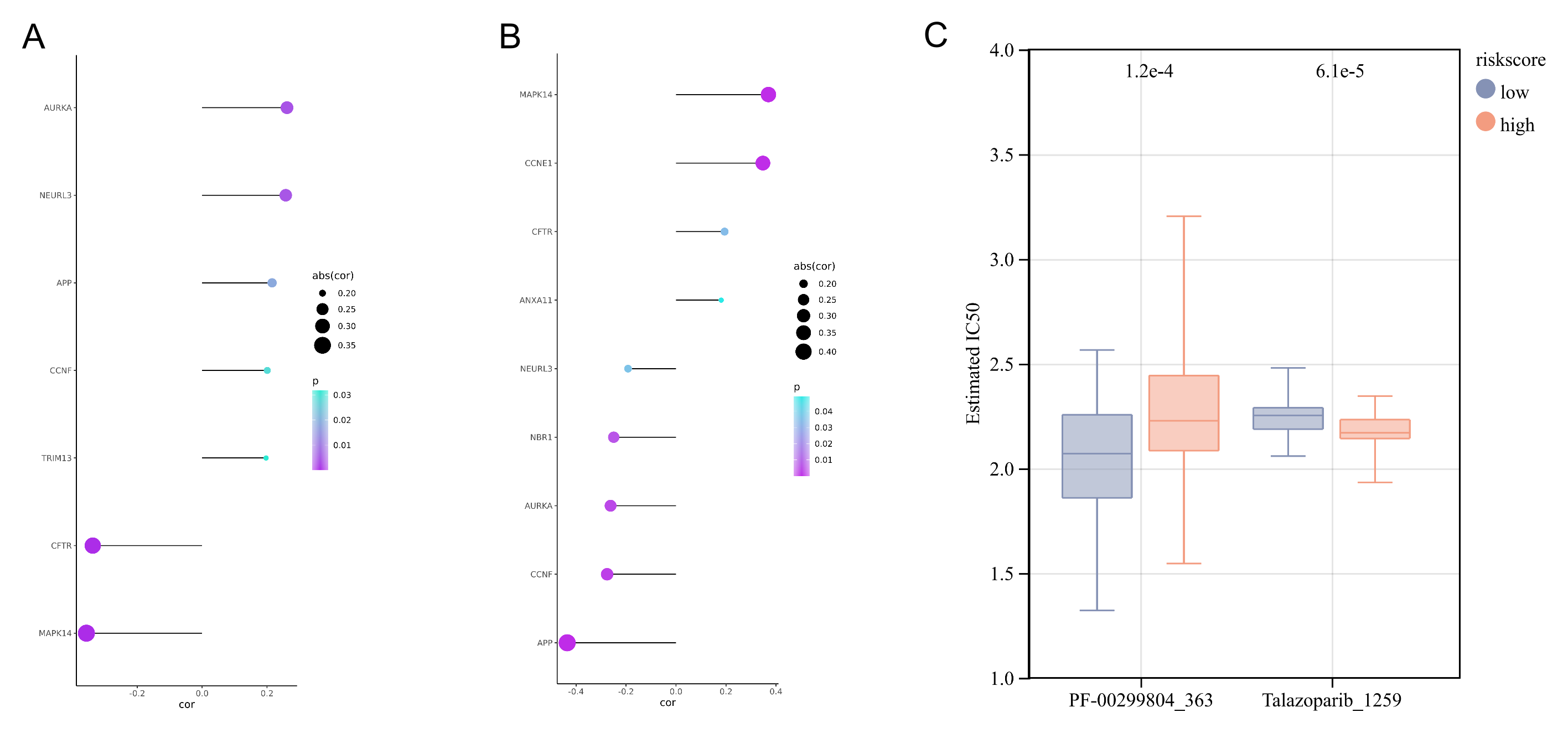


**Fig. S7** (A) Plot of correlation analysis between the sensitivity of dacomitinib and 11 prognostic URGs and DRGs. (B) Plot of correlation analysis between the sensitivity of talazoparib and 11 prognostic URGs and DRGs. (C) The predicted IC50 values of patients from high and low-risk groups on dacomitinib and talazoparib.


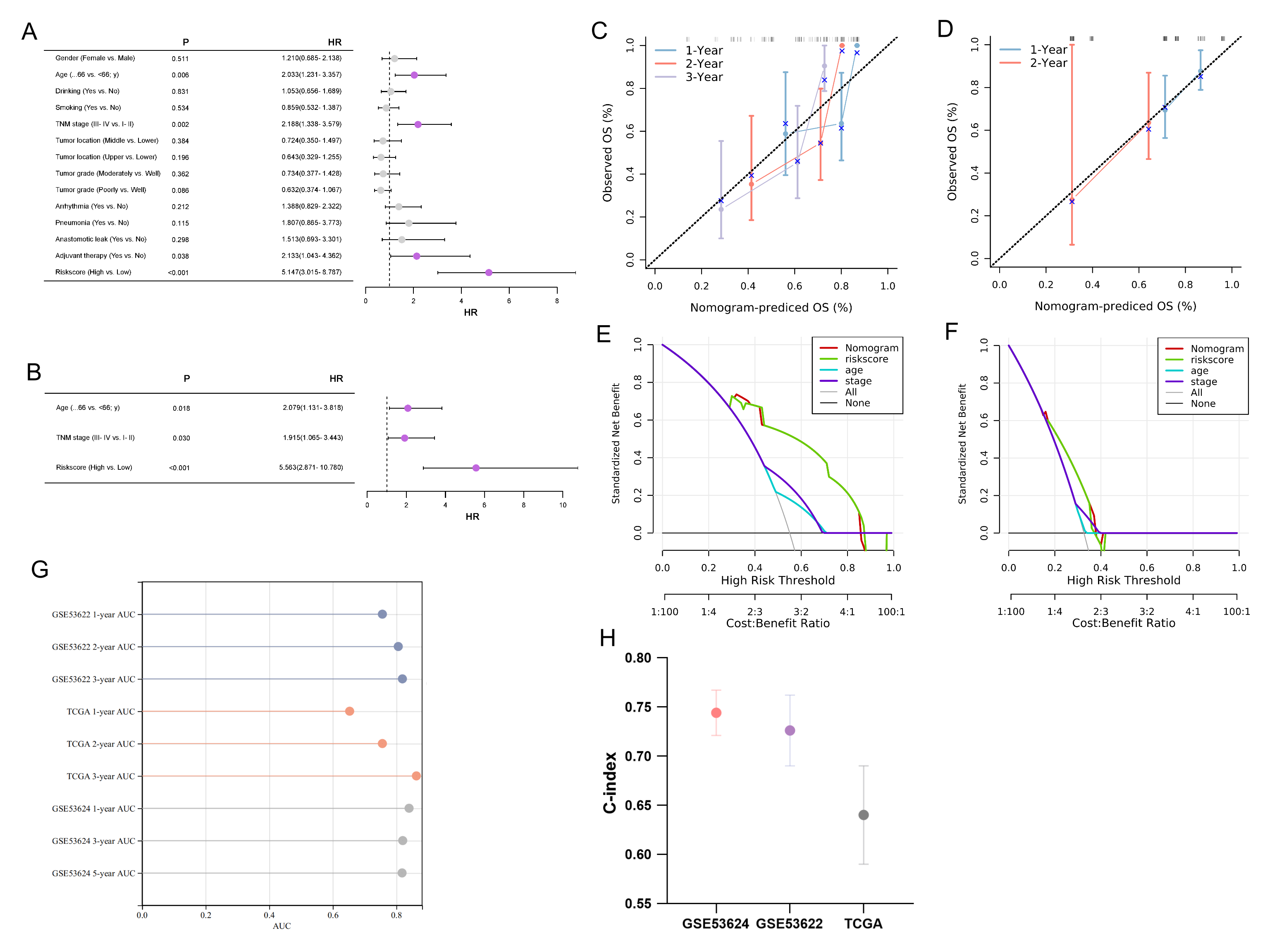


**Fig. S8** (A-B) The HR and 95% CI of potential prognostic clinical parameters and the URGs and DRGs prognosis signature based on the results of univariate (A) and multivariate (B) cox regression analyses. (C) The calibration curve analysis of the nomogram for predicting 1-year, 2-year, and 3-year OS in the GSE53622. (D) The calibration curve analysis of the nomogram for predicting 1-year and 2-year OS in the TCGA-ESCC dataset. (E-F) The DCA curve analysis of the nomogram compared with risk score, age and TNM stage in the GSE53622 (E) and TCGA-ESCC (F) dataset. (G) The time-dependent AUC of the nomogram in the GSE53624, GSE53622 and TCGA-ESCC dataset. (H) The C-index of the nomogram in the GSE53624, GSE53622 and TCGA-ESCC dataset.


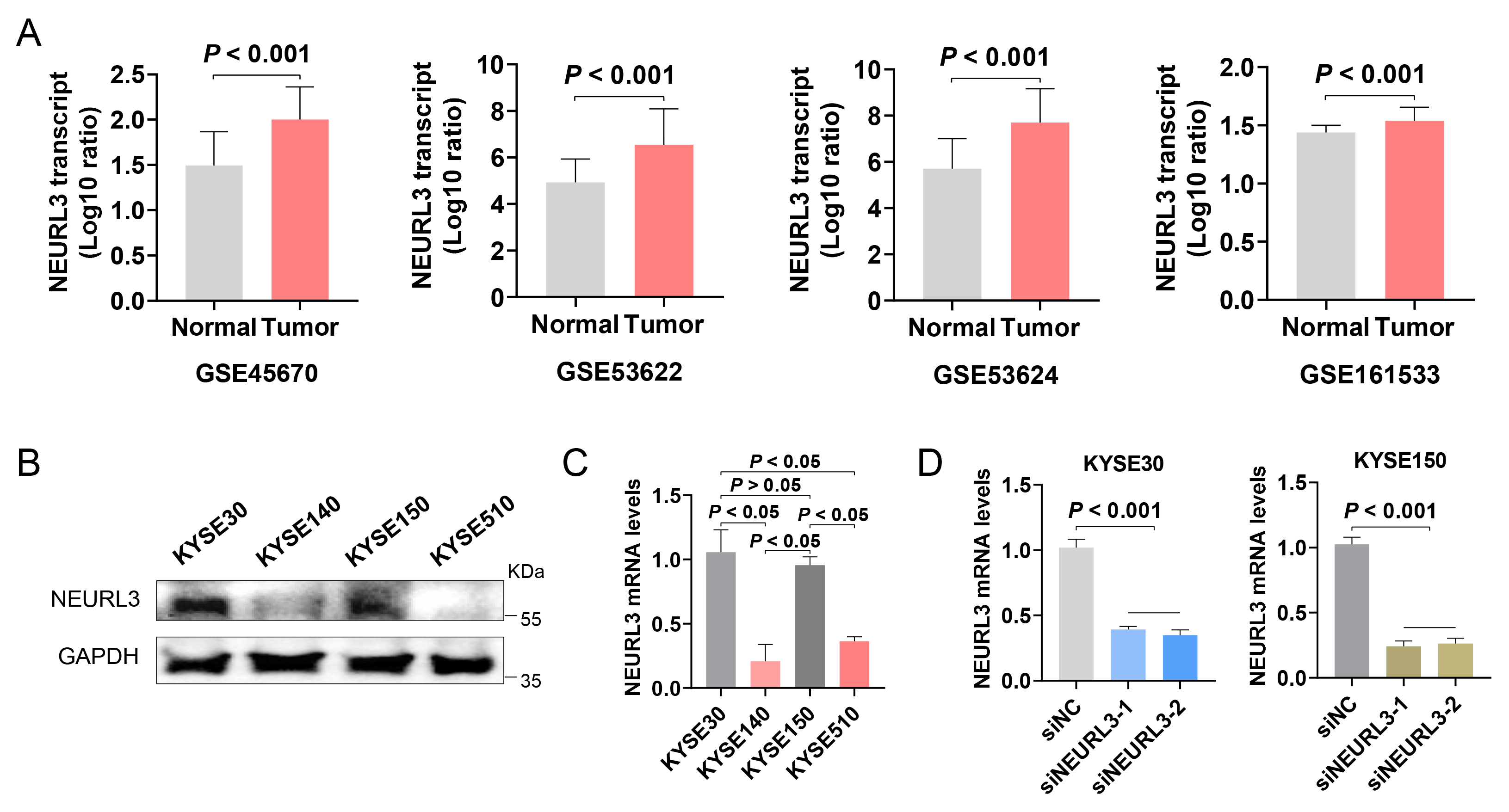


**Fig. S9** (A) The expression level of NEURL3 in ESCC and normal tissues in GEO dataset. (B-C) The expression of NEURL3 in several ESCC cell lines was detected by using Western blotting (B) and RT-qPCR assays (C). (D) RT-qPCR assay was conducted to show knockdown efficiency of NEURL3 in KYSE30 and KYSE150 cells by two independent siRNAs.
